# The wild, wild west of plasmids: First insights into comparative genomics of *Borrelia burgdorferi* sensu lato

**DOI:** 10.1371/journal.pone.0346097

**Published:** 2026-05-08

**Authors:** Sabrina Hepner, Robert E. Rollins, Evangelos Mourkas, Konstantin Kuleshov, Sherwood Casjens, Weigang Qiu, Alexandra Dangel, Dunia Velázquez, Sergey Y. Kovalev, Andreas Wieser, Johannes Hübner, Andreas Sing, Volker Fingerle, Gabriele Margos

**Affiliations:** 1 German National Reference Centre for Borrelia, Oberschleissheim, Germany; 2 Bavarian Health and Food Safety Authority, Oberschleissheim, Germany; 3 Institute of Avian Research “Vogelwarte Helgoland”, Wilhelmshaven, Germany; 4 Zoonosis Science Centre, Department of Medical Sciences, Uppsala University, Uppsala, Sweden; 5 Central Research Institute of Epidemiology, Moscow, Russia; 6 School of Biological Sciences and Division of Microbiology and Immunology, Pathology Department, University of Utah School, Utah, United States of America; 7 Graduate Center and Hunter College, City University of New York, New York, United States of America; 8 Institute of Natural Science and Mathematics, Ural Federal University, Yekaterinburg, Russia; 9 Medical Microbiology and Hospital Epidemiology, Max von Pettenkofer Institute, Faculty of Medicine, LMU Munich, Munich, Germany; 10 Division of Infectious Diseases and Tropical Medicine, LMU University Hospital, LMU Munich, Munich, Germany; 11 German Center for Infection Research (DZIF), Partner site Munich, Munich, Germany; 12 Imunology, Infectious Disease and Pandemic Research (IIP), Fraunhofer Institute for Translational Medicine and Pharmacology (ITMP), Munich, Germany; 13 Dr. von Hauner Children’s Hospital, LMU Munich, Munich, Germany; University of Kentucky College of Medicine, UNITED STATES OF AMERICA

## Abstract

Lyme borreliosis is caused by species of the *Borrelia burgdorferi* sensu lato complex. Genospecies vary in human pathogenicity, reservoir host and tick vector adaptation. What causes these differences is currently unknown, but some answers are likely to be found within the fragmented genomes containing linear and circular plasmids (and even fusions). These plasmids are highly variable and challenging to reconstruct completely. We aimed to analyze plasmid presence/absence and to identify trends in plasmid numbers and/or types associated with specific ecological adaptations or pathogenic potentials. For this, we used the most complete, reliable, and currently available 86 *B. burgdorferi* s.l. genomes (30 newly sequenced, 56 publicly available) belonging to 21 of the 28 known species. The plasmid types per isolate vary from 6-21 (median: 12) and linear plasmid numbers (3–12, median: 7) are significantly higher than circular plasmids (2–11, median: 5). The only plasmids found in all isolates are lp54 and cp26 and only a few species- or population-specific plasmids are identified, demonstrating the high variability. Species with smaller plasmid counts (median < 11) tend to have fewer lp28 and cp32 plasmids than larger plasmid counts species (median ≥ 15). Significantly higher total and circular plasmid numbers are found in rodents-only compared to birds-only associated species. Interestingly, plasmid numbers are not significantly reduced in species associated with a single reservoir host/vector compared to multiple. Analyses of the plasmid-location of the *vls/vlsE* antigenic variation system highlighted the dynamic nature of *B. burgdorferi* s.l. plasmids. Surprisingly, whilst the plasmid repertoire of *B. burgdorferi* s.l. is very plastic and variable, our analyses rarely reveal plasmids associated with specific ecological adaptations or pathogenic potentials. However, some plasmids were more frequently found in one of the groups and will be of interest for gene-based analyses that will be required for further progress in this research area.

## Introduction

Lyme borreliosis is the most frequently reported tick-borne disease in Europe and North America. It is caused by bacteria belonging to the *Borrelia burgdorferi* sensu lato (s.l.) species complex [1,2]. The complex comprises 28 validated and *Candidatus* species (December 2025), varying in human pathogenicity and virulence [2–4]. Six species have been shown to be human pathogens: *B. afzelii, B. bavariensis, B. burgdorferi* sensu stricto (s.s.)*, B. garinii, B. mayonii,* and *B. spielmanii* [1,2,5,6]. The species *B. bissettiae*, *B. lusitaniae*, and *B. yangtzensis* have less frequently been reported to infect humans [7–14], while the species *B. valaisiana* is considered to be nonpathogenic for humans [15]. For most of the remaining species the human pathogenicity is unknown [2,4].

*Borrelia burgdorferi* s.l. are parasitic bacteria that are maintained in natural transmission cycles between tick vectors (*Ixodes* ticks) and various vertebrate reservoir hosts [1,2,16]. Some species can use a wide range of vector species and/or reservoir hosts (generalists), while others are adapted to a narrow range (specialists) [2,16–18]. An example of a generalist species is *Borrelia burgdorferi* s.s. as it can use a wide variety of reservoir hosts (e.g., birds, rodents, insectivores) and vectors (e.g., *I. ricinus, I. hexagonus, I. scapularis, I. pacificus, I. minor, I. affinis*) [2,16]. Other *Borrelia burgdorferi* s.l. species are more specialized and the range of reservoir hosts is narrower, e.g., *B. bavariensis* is adapted to rodents, *B. garinii* is adapted to birds, *B. spielmanii* is adapted to the garden dormouse *Eliomys quercinus* [18,19]. The same is true for tick vector associations, e.g., *B. valaisiana* is adapted to *I. ricinus* while *B. garinii* can be vectored by *I. ricinus*, *I. persulcatus,* and *I. uriae* [16,18]. In general, it is assumed that isolates of the same species share the same ecological niche adaptations [4,18,20], but for some species, distinct populations of the same species may occupy different niches as is known for *B. bavariensis* [17]. The species has been hypothesized to have two populations that are associated with different vectors: The European population of *B. bavariensis* which is considered to be highly invasive in humans [21] is vectored *by I. ricinus*, while the Asian *B. bavariensis* population is associated with *I. persulcatus* [17,22–24]. These adaptations are thought to be driven genetically. Although the genome of *B. burgdorferi* s.l. is small (~1.5 Mbp) it is very unusual for bacteria consisting of a linear chromosome (~ 0.9 Mbp) and numerous linear and circular plasmids (~ 0.6 Mbp) that can constitute up to about 40% of the genome (as in the case of *B. burgdorferi* s.s. strain B31) [25–27]. The chromosome and two plasmids (cp26 and lp54) are universally present and show conserved synteny across species, while the other plasmids are highly variable with regard to presence and gene content [28–31]. The latter is in part due to rearrangements within and between plasmid types and even fusions between different plasmid types [27,28,30–34]. Studies performed mainly on *B. burgdorferi* s.s. strains B31 and 297 have shown that plasmids, genes and sequence sections are important for maintaining the tick-vertebrate reservoir transmission cycle [32–41]. For example, plasmids cp26, lp54, lp25, lp28−1 and lp36 are essential for B31 invasiveness in mice [36,42–50], lp25 and lp28−4 are essential for tick gut colonization, while lp28−1 is important for tick-to-host transmission [47,48,50,51]. In addition, the plasmid encoded proteins listed in Table 1 have been shown to be important. However, many genes involved in adaptation and pathogenicity, especially in species other than *B. burgdorferi* s.s., are not yet fully understood.

**Table 1:** Examples of plasmid encoded proteins that are important for maintaining the tick-vertebrate reservoir transmission cycle.

Protein	Plasmid	Function
OspA	lp54	important for tick colonization by interacting with the tick gut receptor TROSPA [52,53]
OspC	cp26	targets *Borrelia* to tick salivary glands during the blood meal and is important for early stage mammalian infection [54–56]
VlsE	depends on isolate (lp28−1 in *B. burgdorferi* s.s. strain B31)	VlsE outer membrane protein expression site exchanges sequences with the silent *vls* cassettes to generate antigenic variation and is critical for immune evasion and persistent infection [42,43,57–59]
decorin binding proteins (DbpA and DbpB)	lp54	important for mouse infection [60–63]
BBK32	lp36	binds fibronectin and inhibits the classical complement pathway [64–67]
complement regulator-acquiring proteins (CRASPs) and Erps	Examples: lp54 (BbCRASP-1/CspA) lp28−3 (BbCRASP-2/CspZ) cp32s (OspE/F-related proteins)	involved in the evasion of the complement lysis by binding factor H [37,46]
intergenic spacer (317 bp long)	lp17	involved in the colonization of mouse tissue and the induction of carditis and arthritis [68]

The assembly of *Borrelia* complete genome sequences is challenging due to the high number of plasmids (up to 24, often with high sequence similarity, such as the cp32 and lp28 plasmid families), the variable plasmid repertoire, and the mosaic structure of plasmids, thereby requiring robust *de-novo* sequence assembly strategies [26,28]. Long read sequencing and a combination of several assembly strategies have greatly improved *Borrelia* genome reconstruction and currently enables the most complete *Borrelia* plasmid sequences to be determined [69–73]. These technological advances have opened the door for more sophisticated analyses of plasmids and plasmid encoded genes. While early comparative genome and plasmid analyses have included draft genomes based on short-read or shotgun Sanger sequencing [29–31,74,75], recent work has analyzed high-quality genomes based on long read sequencing data, although, with a focus on conserved genomic compartments, such as the chromosome, and plasmids lp54, cp26, and lp17 [72].

In this study, we used the most complete *B. burgdorferi* s.l. genomes currently publicly available (n = 56), and we present 30 new genome sequences of *B. bavariensis, B. garinii* and *B. valaisiana* isolates based on long reads sequence data and a high-fidelity approach for *Borrelia* genome reconstruction [73]. The dataset includes 21 *B. burgdorferi* s.l. species with different reservoir host and vector associations as well as different human pathogenicity potentials. As a first step in identifying molecular trends for ecological adaptations and pathogenicity, we created a plasmid presence/absence matrix for the 86 isolates, and we present here an analysis of plasmid numbers and types based on species, reservoir host association, vector association, and human pathogenicity.

## Materials and methods

### Samples

We report high-quality genome sequences of 30 *B. burgdorferi* s.l. isolates belonging to the species *B. bavariensis*, *B. garinii*, and *B. valaisiana* that are marked with an asterisk (*) in Table 2 (see strain details in S1 Table). The newly sequenced isolates are from the collection of the German National Reference Centre for *Borrelia* (n = 28) or DNA provided by Sergey Y. Kovalev (n = 2; Prm7564-11 and Tmsk976–2013). These species were chosen as they showed both differences and similarities in their ecological adaptations and human pathogenicity, providing a good basis for comparative genome and plasmid analyses.

**Table 2:** Genomes used in this study. Dataset consists of 86 isolates belonging to 21 *B. burgdorferi* s.l. species. Newly sequenced isolates are marked with an *. #: according to reports by Postic et al. 2007 [76] and Margos et al. 2009 [77], isolates of this clade were used to determine species borders and were considered *B. burgdorferi* s.s. by those authors based on DNA-DNA hybridization and MLST analyses.

Species	Number of isolates	Isolates
*B. americana*	2	SCW-30h, SCW-41
*B. andersonii*	2	21038, MOD-5
*B. bavariensis*	21	A104S*, A91S*, DK6*, Lubl25*, NT24*, PBaeII, PBar*, PBi, PBN*, PEi*, PHerI*, PLad*, PNeb*, PNi*, PRab*, Prm7564-11*, PRof*, PTrob*, PWin*, PZwi*, Tmsk976–2013*
*B. bissettiae*	1	PGeb
*B. burgdorferi* s.s.	16	217_5, 80a, B31, Bol29, Fr-93–1, NE5248, NE5261, NE5267, NIH3, NIH5, NIH8, PAbe, PAli, SCW-9, Sh-2–82, Z9
*B. californiensis*	2	CA443, CA446
*B. carolinensis*	5	BUL-H-2, M7p, MI-3, SCGT-18, SCW-22
*B. finlandensis* *(B. burgdorferi* s.s.#*)*	1	Z11
*B. garinii*	12	20047, NT31*, PBes, PBr*, PFr*, PHC*, PHei*, PKi*, PLa*, PLi*, PMa*, PMe*
*B. japonica*	2	HO14, Miyazaki2E
*B. kurtenbachii*	1	25015
*B. lanei*	1	CA-28–91
*B. lusitaniae*	3	PoHL1, PotiB2, PotiB3
*B. maritima*	1	CA690
*B. mayonii*	2	MN14–1420, MN14–1539
*B. sinica*	1	CMN3
*B. spielmanii*	1	PMew
*B. tanukii*	4	Hk501, Koshiki4E, TanegashimaAS13, TanegashimaAS9
*B. turdi*	2	047−3, Ya501
*B. valaisiana*	3	100B40*, 89B13 Am501
*B. yangtzensis*	3	Miyako4E, Okinawa-CW61, Okinawa-CW62
**total: 21 species**	**total: 86 isolates**	

The additional 56 publicly available *B. burgdorferi* s.l. genomes (accession numbers in S1 Table) used here were re-checked for completeness (see the section ‘Completeness check’ below). In total 86 genomes belonging to 21 *B. burgdorferi* s.l. species were included in this study. All analyzed genomes were sequenced using long read sequencing technology (Pacific Biosciences) and represent high-quality genome assemblies.

### Assembly of new genome sequences

We assembled 26 genomes of the 30 newly sequenced *B. burgdorferi* s.l. isolates using the high-fidelity approach described by Hepner et al. 2023 [73] (assembly statistics are shown in S1 Table of Hepner et al. 2023 [73]). This approach included *Borrelia* culturing, DNA extraction, nucleotide sequencing (PacBio long read and Illumina short-read technology), assembly (PacBio data: microbial genome assembler (MGA) (SMRT Link v8.0.0.80529), Improved Phase Assembler (IPA) (SMRT Link v10.1.0.115488), and HiCanu [78]; Illumina data: SPAdes [79]; PacBio and Illumina data: hybridSPAdes [79,80]), quality check (QC) and refinement steps, and the generation of a final combined consensus (a schematic overview is shown in S1 Fig). The QC and refinement of the PacBio assemblies included assembly statistics, contig trimming and the reconstruction of the genome. Plasmids were typed based on the PFam32 plasmid partition gene loci if present [28,30–32,81]. Duplicates and misassembled contigs were deleted. If plasmids were split in multiple contigs, sequences were merged at sequence overlaps. Every genome element was checked for completeness (see below). To confirm the plasmid composition of isolates, SPAdes assemblies (and for complicated genomes also hybridSPAdes assemblies) were additionally analyzed for presence of PFam32 genes. For each isolate the assembly results were manually compared regarding correctness and completeness and sequences were combined to generate a final combined consensus as complete as possible. As a modification to the process described in Hepner et al. 2023 [73], the MGA assemblies (based on continuous long reads (CLR) generated by the PacBio Sequel instrument) were not polished with Illumina reads due to improved PacBio CLR accuracy (in comparison to CLR generated by older PacBio instruments as the PacBio RS II).

The remaining four isolates (A104S, NT31, NT24, PHei) were sequenced using an older PacBio RS II instrument, resulting in CLR with lower accuracy. These were assembled using HGAP v3 (Pacific Biosciences, SMRT Analysis Software v2.3.0) (S1 Table). For circularization, the Minimus2 software of the Amos package was used. The RS_Resequencing.1 software (SMRT Analysis version v2.3.0) was used to map reads back to assembled and circularized sequences in order to correct sequences after circularization. To polish indels and sequencing errors that may be present in the assemblies, Illumina short reads were mapped to the HGAP contigs scaffold and a consensus sequence was extracted using CLC Genomic Workbench v.21. The following stringency settings were used for read mapping and consensus extraction: match score 1, mismatch cost 2, insertion and deletion cost 3, similarity fraction 0.8 and length fraction 0.9. If no Illumina reads matched, the consensus sequence was filled from the HGAP contig and as conflict resolving strategy “vote” was used. The QC and refinement steps and the generation of the final consensus were conducted according to Hepner et al. 2023 [73]. For the isolate NT24, DNA was also extracted with the Nanobind CBB Big DNA Kit (Circulomics, USA) (as described in Hepner et al. 2023 [73] for isolate 89B13) and sequenced on a PacBio Sequel II instrument.

### Completeness check of GenBank genomes

The 56 publicly available genomes were checked for completeness as follows: Plasmids were considered complete when terminal wraparound sequence and “properly” spaced 14-bp TAGTATA telomere motifs (S. Casjens, to be published elsewhere) were present in linear plasmids, or terminal direct repeats were found in circular plasmids [73,82]. Wraparound sequences and terminal direct repeats were identified by aligning the contig with itself using a web-based nucleotide NCBI-BLASTN search with default parameters using the setting “align two or more contigs” [83]. The telomere motif analyses were conducted manually. Details are described in the methods section of Hepner et al. 2023 [73].

Three of the genomes (PBaeII, PBes, and 89B13) were previously published by Hepner et al. 2023 [73]. Four genomes (PBi and 20047 [84], PAbe and PAli [85]) were also sequenced by our team and were re-analyzed for correct trimming and completeness and were corrected as needed. Additionally, SPAdes assemblies were searched for presence of PFam32 genes and to confirm the completeness of plasmid composition. The genome of *B. burgdorferi* s.s. strain B31 [25] available in GenBank is missing the end sequences of the linear plasmids (including the 14-bp TAGTATA telomere motif); these were kindly provided by George Chaconas (University of Calgary, Canada) and some were reported by Tourand et al. 2009 [86], Huang et al. 2004 [87], and Hinnebusch et al. 1991 [88]. The linear genome elements of MN14–1420 and MN14–1539 [69] were checked for the 14-bp TAGTATA telomere motif (and if found, were considered as complete), and circular plasmid completeness was assumed. The remaining 46 genomes were sequenced by Akther et al. 2024 [72] and completeness status of each genome element is listed in S2 Table of that manuscript.

### Plasmid names and fusion plasmids

Plasmids were typed and named according to their PFam32 loci by comparing query sequences to previously published PFam32 loci, using thresholds of  ≥ 86% nucleotide identity and ≥ 90% subject coverage and BLAST v.2.2.31 with the algorithm BLASTN and default settings [83,89] (details are described in Hepner et al. 2023 [73], PFam32 accession numbers are listed in S2 Table). If PFam32 loci were identified as fragments or pseudogenes, these were also included if they matched the defined thresholds. If more than one PFam32 locus was present that matched the defined thresholds, plasmids were considered to be fusions. The publicly available genomes were re-checked for the presence of PFam32 loci and names were edited if needed so that the present PFam32 are clearly identifiable by the names. S3 Table lists the plasmids with edited names including information about the published names, accession numbers, and plasmid names used in this study.

### Definition of ecological adaptations and pathogenicity status

The following publications and references cited therein were used to define reservoir host and vector associations, and human pathogenicity status: Margos et al. 2023 [2], Margos et al. 2022 [4], Eisen 2020 [16], Wolcott et al. 2021 [18]. The groups of ecological adaptations and pathogenicity status are presented in the results chapter “Plasmid variation among different ecological niche adaptations (reservoir host and vector) and pathogenicity groups” (for details see S1 Table).

The species *B. bavariensis* are containing two genetically distinct populations (European and Asian) [24,90] that differ in the ecological niche adaptation and were analyzed separately. *Borrelia bavariensis* is supposed to have undergone a host switch followed by a vector switch leading to increased virulence and it is known that the plasmid repertoire of the two populations differs [17,21,33,91]. This makes the species highly interesting aiming to analyze plasmid patterns associated with ecological niche adaptation (reservoir host and vector) and human pathogenicity [17,24,33,77,90]. Both populations were categorized as rodents-adapted, while the Asian population is primarily associated with *I. persulcatus* and the European populations is exclusively found in *I. ricinus* ticks and show increased invasiveness in humans [22,23,90].

### Phylogenetic analyses

Phylogenetic analyses of the 86 genomes were conducted based on 639 cgMLST loci [92]. The MAFFT [93] alignment of the concatenated cgMLST sequences was generated on the *Borrelia* PubMLST website using the “Genome Comparator” [94] while gaps due to missing nucleotides, incomplete loci or missing loci were indicated by “-“ in the alignment. A maximum likelihood (ML) tree was generated using IQ-TREE 2.2.2.7 [95], 1000 ultrafast bootstrap replicons (UFBoot) [96] and the substitution models GTR+F+I+R7 (ModelFinder was used for model selection as part of IQ-TREE [95,97]). In IQ-TREE, missing characters and gaps (“-” in the alignment) are treated as unknown states and are accommodated in the likelihood calculation without removing alignment sites, so that all available information from other samples at each position is retained. Tree visualization was conducted in the online tool iTOL (Interactive Tree of Life) v7 [98].

### *vls/vlsE* location

As it is known that the *vlsE* loci and the silent *vls* cassettes are especially challenging to assemble and to annotate [71,99,100], we paid special attention to this locus the see whether it is successfully reconstructed and on which plasmid it is located in the different isolates. For this, genomes were aligned with *vls/vlsE* reference sequences using a web-based nucleotide NCBI-BLASTN choosing the option “somewhat similar sequences” [83]. *Vls/vlsE* sequences from *B. burgdorferi* s.s. B31 (lp28−1: NC_001851.2; sequences: WP_044283658.1, WP_052403612.1, WP_257730674.1, WP_164928172.1), *B. garinii* 17-58N4 (lp28−9: NZ_CP118302.1, sequences: OQ450376.1, OQ450377.1), and *B. japonica* HO14 (lp28−8: CP179486.1, XPC97921.1) were used as references.

### Statistical diagrams and analyses

Statistical analyses were conducted using R v4.4.1 and RStudio 2024.04.2 [101]. Boxplots, barplots and probabilities were visualized using the package ggplot2 [102]. For plasmid presence, we estimated 95% confidence intervals (CI) using the Clopper-Pearson method (command: *exactci*) in the package PropCIs [103]. Additionally, descriptive statistics were computed including the count (total number of observations), median (m, middle value), mean (average), first quartile (Q1, the value below which 25% of the data fall), third quartile (Q3, the value below which 75% of the data fall), interquartile range (IQR = Q3 – Q1, range containing the central 50% of the data), minimum (min, smallest value), and maximum (max, largest value).

We ran generalized linear mixed effects models (GLMM) using the function *glmer* from the R-package *lme4* [104] with a Poisson error distribution to estimate total, circular, and linear plasmid numbers in relation to reservoir host association groups (single, multiple, unknown), vector association groups (single, multiple, unknown), and human pathogenicity groups (pathogenic, potentially pathogenic, nonpathogenic, unknown) which were included as fixed effects. Here we included genospecies as a random effect to correct for species-specific differences. Isolates with the host class “single?” (n = 4) were removed from this analysis due to low sample size and uncertain status. A second GLMM using a Poisson error distribution was also run on the subset of birds-only and rodents-only associated isolates, with host association (birds-only *vs* rodents-only) being taken as a fixed effect and genospecies as a random effect. For both models, mean estimates and their 95% credible intervals (CIs) were estimated based on 10000 simulations using the function *sim* from the package *arm* [105] and were considered significant if the 95% CI did not overlap with 0. Residual errors were calculated according to Nakagawa et al. 2010 [106]. We additionally performed Kruskal-Wallis-tests using the *stat_compare_means* command from the package ggpubr [107] to test differences between groups of the same plasmid topology (differences between groups were found if p < 0.05). This was followed by a pairwise Wilcoxon-test with a Bonferroni multiple testing correction using the *pairwise_wilcox_test* command from the package rstatix [108], resulting in adjusted p-values (p.adj). The levels of statistical significance were symbolically represented in the boxplots: p.adj ≥ 0.05: “ns” not significant, p.adj < 0.05: “*” significant, p.adj < 0.01: “**” highly significant, p.adj < 0.001: “***” very highly significant, p.adj < 0.0001: “****” extremely significant. This was done to include all groups even if sample sizes were low or not applicable for GLMM analysis.

Plasmid co-occurrence was analyzed using the R-package cooccur [109]. An expected co-occurrence was calculated followed by a check if the observed value deviates (positive, negative) or is not different (random). Here plasmid types were taken as “species” with individual isolates being considered “patches”. We ran the analysis individually on all isolates (n = 86) and subsets of the data for pathogenic isolates (n = 52), single vector association (n = 47), multiple vector association (n = 37), single host association (n = 60), multiple host association (n = 20), birds-only associated (n = 17), and rodents-only associated (n = 40).

### Ethical statement

This study did not involve human participants or human experimentation. Only bacterial isolates from an existing laboratory strain collection were analyzed; no new samples were collected for this study. The published genomic data and associated metadata relate exclusively to bacterial characteristics and do not allow identification of individual patients. Therefore, ethical approval and informed consent were not applicable.

## Results

### Challenges of *B. burgdorferi* s.l. genome assembly and analyses

#### Assembly of 30 new genomes resulted in high-quality *B. burgdorferi* s.l. genomes.

Complete genome sequences of *B. burgdorferi* s.l. have been challenging to determine due to the presence of many plasmids that harbor numerous repeated sequences. In this study, we report genome sequences of 30 *B. burgdorferi* s.l. isolates (19 *B. bavariensis,* 10 *B. garinii*, one *B. valaisiana*), and we confirm that the high-fidelity approach described by Hepner et al. 2023 [73] and here in Materials and Methods is well suited for high-quality *B. burgdorferi* s.l. genome sequence determination if sufficient DNA of appropriate quality is used. An overview of the workflow is shown in S1 Fig, and details are described in the Material and Methods section.

The 30 newly sequenced genomes contain 384 genome elements: 30 chromosomes and 354 plasmids (230 linear and 124 circular plasmids that include 37 fusion plasmids that carry more than one PFam32 gene; S1 Table). No new plasmid PFam32 types were found. For the majority of the genome elements (n = 161) the HiCanu assembler reconstructed them most completely and correctly and therefore the HiCanu contig is mostly used in the final combined consensus, followed by MGA (n = 113), HGAP (n = 48), IPA (n = 47), and (hybrid)SPAdes (n = 6, some plasmid sequences found only in the MiSeq data); some genome elements were reconstructed by merging contigs produced by different assemblers (n = 9) (S1 Table includes assembly information for each genome, S4 Table shows an overview). Out of 384 genome elements, 364 genome elements are completely reconstructed while only 20 failed to completely assemble due to a lack of end sequence in linear plasmids or lack of overlap in circular plasmids (S1 Table).

Genomes are considered as complete if all the identified genome elements are completely reconstructed, indicated by terminal wraparound sequence and telomere motifs in the untrimmed chromosome and linear plasmids or terminal direct repeats in the untrimmed circular plasmids. Using the high-fidelity approach (S1 Fig), we succeeded in determining the complete genome sequences of 18 of the 30 genomes reported here (S1 Table). In a further seven genomes only a single plasmid (6x lp28–8, 1x lp28–4) is not fully reconstructed and in two genomes SPAdes contigs with *vls/vlsE* fragments that are not present in the PacBio data were found. In the remaining three samples (Prm7564-11, Tmsk976–2013, PHei) two or more plasmids are not fully reconstructed. The incomplete plasmid sequences are mainly cp32 (n = 7) and lp28 (n = 11) plasmid families that are known to be challenging to assemble. We note that different members of the cp32 family can have long several kbp regions that are nearly identical, and this also causes problems with sequence assembly [26,28,34,38,110]. The sequence of the region that encodes the VlsE surface lipoprotein and its associated *vls* cassettes proved to be the most difficult to assemble and this is often present on lp28s (see next section).

### *Vls/vlsE* analyses

The antigenic variation of *B. burgdorferi* s.l. is based on the *vls/vlsE* system [57,58]. It consists of a single expression locus (*vlsE*) and – depending on the isolate – about 16 quite similar silent *vls* cassettes [99]. The expressed *vlsE* gene can be modified in situ by random recombinational replacement with segments of the *vls* cassettes, leading to the evasion of the reservoir host adaptive immunity and permitting a persistent *B. burgdorferi* s.s. infection [42,43,57–59,111].

The *vls/vlsE* system is located on lp28s in many isolates, and the lp28–8 plasmid with its *vls/vlsE* region is incompletely assembled in seven newly sequenced isolates due to poor HGAP assembly from PacBio data (NT24, A104S and PHei) or due to being present only in short SPAdes contigs (A91S, DK6, PRof, PWin). In six of these, all but PHei, the only incomplete aspect of the genome is the *vls/vlsE* carrying plasmid. In addition, in three of the *B. bavariensis* genomes reported here (PBN, PZwi, and Tmsk976–2013) the *vls/vlsE* system sequence is found only on small SPAdes contigs (length 0.3–9kbp) that do not have a PFam32 locus.

The *vls/vlsE* region is found on several different plasmids in *B. burgdorferi* s.l. isolates [25,30–32] and is present in 70 of the 86 genomes analyzed here. In a majority of these isolates (n = 61), the *vls/vlsE* system is located on plasmids of the lp28 family (lp28–1 (n = 2), lp28–1 + 11 (n = 4), lp28–3 (n = 18), lp28–8 (n = 36), lp25 + 28–8 (n = 1)), while in six others it is found on lp25 (n = 1), lp36 (n = 4), and lp32–7 (n = 1) (Table 3 and S1 Table). In *B. burgdorferi* s.s. type strain B31 the *vls/vlsE* is located on plasmid lp28–1 + 11 (named lp28–1 in previous publications, S3 Table) [112]. Interestingly, in seven analyzed *B. burgdorferi* s.s. isolates (including B31) the plasmid lp28–1 or lp28–1 + 11 is present, but only in six of these isolates the *vls/vlsE* system is located on this plasmid (Table 3 and S1 Table). The remaining eight *B. burgdorferi* s.s. isolates carry the *vls/vlsE* on lp28–3 or lp28–8 (Table 3 and S1 Table). For three *B. bavariensis* isolates the *vls/vlsE* system sequence was found only on small SPAdes contigs (PBN, PZwi, and Tmsk976–2013; see previous paragraph, “unknown” in Table 3 and S1 Table). The *vls/vlsE* system is not found in the genome assemblies of 16 isolates (“missing” in Table 3 and S1 Table).

**Table 3:** Number of *vls/vlsE* carrying plasmids per species. unknown: *vls/vlsE* was found on SPAdes contigs with missing PFam32 loci; missing: *vls/vlsE* was not found in the genome assembly.

Species (Number of isolates)	*vls/vlsE* location									
	lp28−1	lp28−1 + 11	lp28−3	lp28−8	lp25 + 28−8	lp25	lp36	lp32−7	Unknown	Missing
*B. americana* (n = 2)				1			1			
*B. andersonii* (n = 2)					1		1			
*B. bavariensis* EU (n = 18)				15					2	1
*B. bavariensis* AS (n = 3)			1	1					1	
*B. bissettiae* (n = 1)			1							
*B. burgdorferi* s.s. (n = 16)	2	4	5	3						2
*B. californiensis* (n = 2)										2
*B. carolinensis* (n = 5)			2			1				2
*B. finlandensis* (n = 1)							1			
*B. garinii* (n = 12)			7	3				1		1
*B. japonica* (n = 2)				2						
*B. kurtenbachii* (n = 1)			1							
*B. lanei* (n = 1)				1						
*B. lusitaniae* (n = 3)				3						
*B. maritima* (n = 1)							1			
*B. mayonii* (n = 2)				2						
*B. sinica* (n = 1)										1
*B. spielmanii* (n = 1)				1						
*B. tanukii* (n = 4)				1						3
*B. turdi* (n = 2)			1	1						
*B. valaisiana* (n = 3)				2						1
*B. yangtzensis* (n = 3)										3
**Total (n = 86)**	**2**	**4**	**18**	**36**	**1**	**1**	**4**	**1**	**3**	**16**

### Overview of ecology and genomes

**Fig 1:**
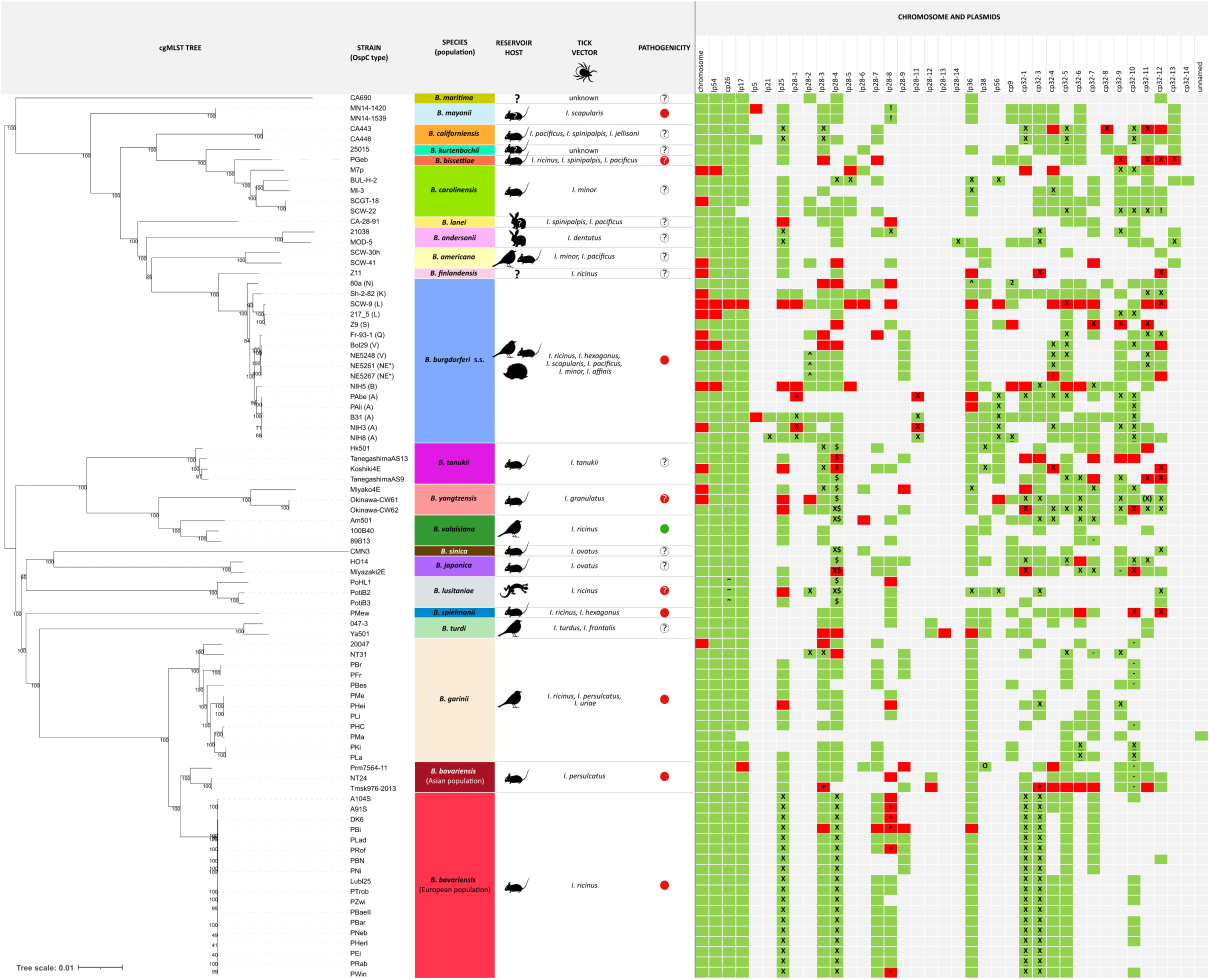
Maximum likelihood tree of the 86 genomes based on cgMLST and plasmid presence/absence matrix. Figure includes information about species, reservoir host, tick vector, pathogenicity and genome elements. Species are listed and colored in different colors. Reservoir hosts are shown as icons; unknown hosts are indicated by “?”; for insectivores and carnivores a hedgehog icon is used. Tick vector species are listed. Pathogenicity is shown in different colors (green: nonpathogenic, red: pathogenic, red and “?”: potentially pathogenic, white and “?”: unknown). Genome elements (chromosome and plasmids): presence is indicated in green (complete genome element) and red (incomplete genome element), absence is indicated in grey; “X”: fusion plasmid, “-”: linear plasmid that carry a PFam32 that is typically found on a circular plasmid, “O”: circular plasmid that carry a PFam32 that is typically found on a linear plasmid. “$”: cp32-28-4 (circular plasmid with syntenic gene contents to cp32 plasmid family but encodes a lp28−4 type PFam32 protein), “^”: inverted repeat plasmid, “~”: dimer fusion, “+”: sequence was additionally added, “2”: two cp9 plasmids with different PFam57 proteins. “(X)”: the same cp32−11 type PFam32 was found on a non-fused plasmid (cp32−11) and on a fusion plasmid (cp32−9 + 11); “!”: plasmid name used in this study differs to the published plasmid name (S3 Table). S2 Fig adds information about the continent, *vls/vlsE* located plasmid, fusion plasmids, and shows reservoir host and vector not as icons, but in separate color-coded columns. S1 Table presents this information in tabular form with additional information on plasmid length.

The panel of 86 high-quality genomes belong to 21 species in the *B. burgdorferi* s.l. complex and have various reservoir host associations (birds, rodents, insectivores, carnivores, lagomorphs, and lizards), variable vector associations (sixteen *Ixodes* species), differing human pathogenicity (pathogenic, potentially pathogenic, nonpathogenic, unknown pathogenic potential) and even differing pathogenicity levels (Asian *B. bavariensis*: localized infection, European *B. bavariensis*: disseminated infection) [17,21]. Information about reservoir host, vector, and pathogenicity are shown in Fig 1 and S1 Table.

The 639-loci cgMLST ML chromosomal tree of the 86 isolates was constructed as described in Hepner et al. 2025 [92] (Fig 1). It confirms and extends chromosomal trees of previous studies [29,72,90,92]. It shows the previously known clustering according to species and grouping into a clade that originated in Eurasia (*B. bavariensis*, *B. garinii*, *B. japonica*, *B. lusitaniae*, *B. sinica*, *B. spielmanii*, *B. tanukii*, *B. turdi*, *B. valaisiana*, *B. yangtzensis*) and a clade that originated in North America (*B. americana*, *B. andersonii*, *B. bissettiae*, *B. burgdorferi* s.s., *B. californiensis*, *B. carolinensis*, *B. finlandensis*, *B. kurtenbachii*, *B. lanei*, *B. maritima*, *B. mayonii*). We note that *B. bissettiae* and *B. burgdorferi* s.s. also occur in Europe and *B. finlandensis* (*B. burgdorferi* s.s.) has only been found in Europe. As expected, the complete chromosomes of all have similar sizes, ranging from 901,026–915,327 bp. Intriguingly, we did not find an obvious distribution pattern of plasmid types among the isolates, not even for isolates of the same species (Fig 1 and S2 Fig, S1 Table). Furthermore, also isolates of the genetically very homogeneous European *B. bavariensis* population (light red in Fig 1 and S2 Fig) do not have identical plasmid contents. This analysis confirms and extends conclusions drawn by previous studies with a lower number or less accurate genomes [31,33,72].

### Plasmid types and numbers

**Fig 2:**
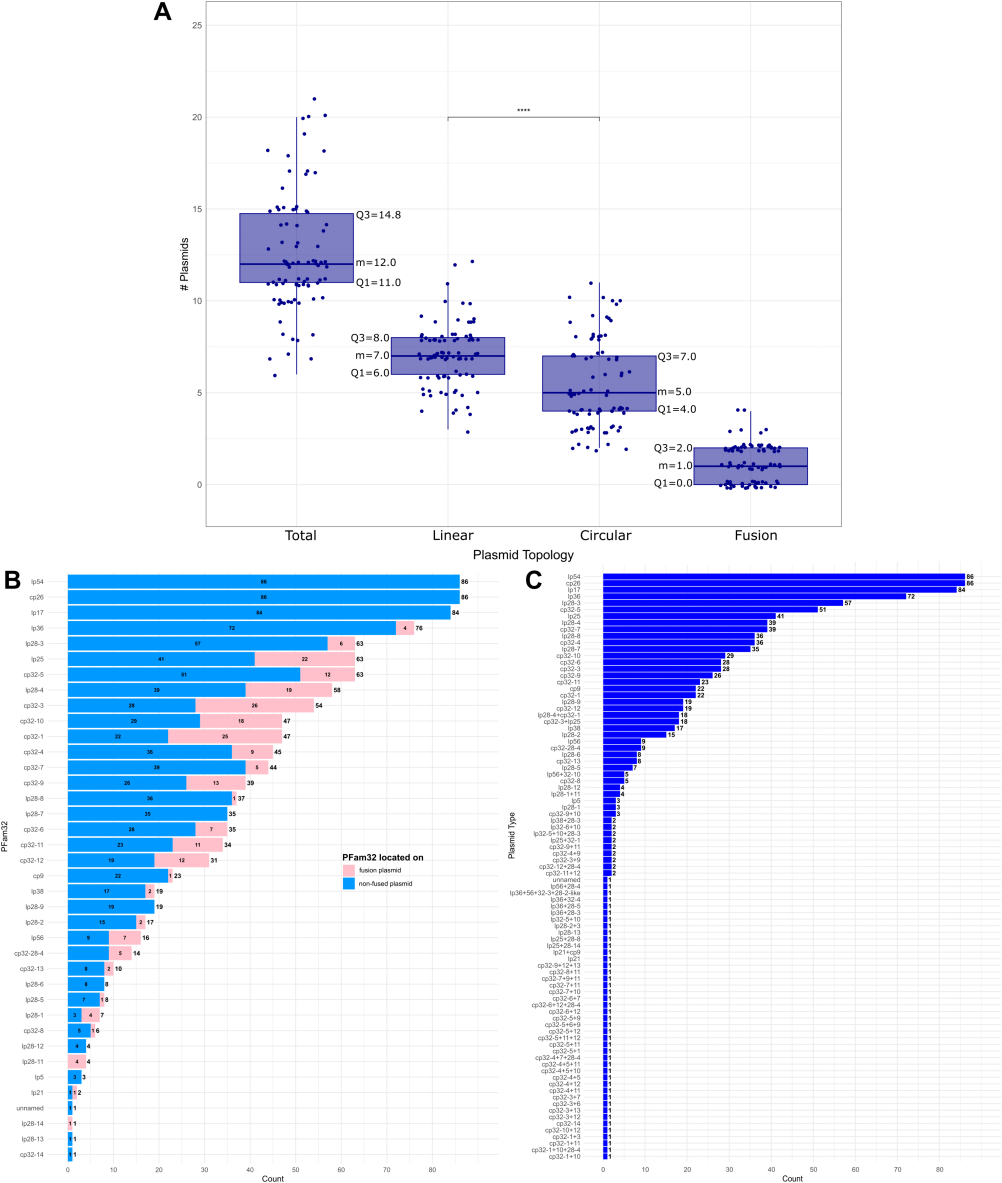
Plasmid numbers per topology and plasmid types. **(A)** Plasmid number range based on plasmid topology (total, linear, circular, fusion). The category total plasmids includes linear, circular, and fusion plasmids. The categories linear and circular plasmid numbers include fusion plasmids. Result of pairwise Wilcoxon-test (linear *vs* circular) are shown: “****” very highly significant: p.adj = 1.98E-06. Information about the median values (m), Q1 and Q3 is included. **(B)** Prevalence of PFam32. The length of the bars corresponds to the count of the found PFam32 loci in the dataset and the exact number is shown at the right tip of the bar. Different PFam32 may be present on the same plasmid in case of fusion plasmids. Bars are colored according to the PFam32 loci location on none-fused plasmids (blue) or on fusion plasmids (pink) and corresponding counts are shown as numbers in the colored bar. **(C)** Prevalence of plasmid types including the actual fusion plasmids. The length of the blue bars corresponds to the prevalence of the plasmid type in the dataset and the exact number is shown at the right tip of the blue bar.

The 86 genomes in our panel contain 1076 plasmids (611 linear plasmids and 465 circular plasmids). The number of linear plasmids was significantly higher than circular plasmids (adjusted p-values (p.adj) = 1.98E-06, “****”; see Materials and Methods for statistical calculations) (Fig 2A). The total number of plasmids in the various isolates ranges from six (*B. lusitaniae* PoHL1) to 21 (*B. burgdorferi* s.s. B31) with a median value (m) for the whole panel of 12.0 (the first quartile (Q1) is 11.0 and the third quartile (Q3) is 14.8) (Fig 2A, S5 Table). The linear plasmid numbers range from 3 to 12 (m = 7.0, Q1 = 6.0, Q3 = 8.0) per isolate and circular plasmid numbers vary from 2 to 11 (m = 5.0, Q1 = 4.0, Q3 = 7.0).

In the 86 isolates, all known 35 PFam32 plasmid types are found (Fig 2B, S1 Table) (known PFam32 types: see S2 Fig in Akther et al. 2024 [72]). Twenty of these PFam32 type genes are commonly found on linear plasmids, 15 are commonly found on circular plasmids including cp9 that carries a PFam32 gene. Additionally, cp9 and lp5 that lack the PFam32 gene but carry the PFam57 gene were found, including the two different types cp9−1 and cp9−2 that can co-exist in one sample [72]. The newly sequenced isolate PMa *B. garinii* also has one complete linear plasmid that is lacking the PFam32 gene but carries a PFam57 and is designated “unnamed” (*B. garinii* PMa).

As mentioned above, many *B. burgdorferi* s.l. plasmids appear to have undergone past rearrangements. A strong indicator of such rearrangements is the fusion of two different plasmid types, and these events can be reflected by the presence of more than one (and up to four) type PFam32 partition/compatibility type gene on a single plasmid. This is of course a very minimal estimate of past plasmid fusions since events that have parental parts that lack a PFam32 gene are not recognized as fusions. Additionally, PFam32 pseudogenes that do not match our stringent thresholds (see Materials and Methods) were not considered as fusion plasmid indicators.

When plasmids with the same plasmid topology (linear or circular) fuse, the resulting fusion plasmid should retain the same topology, while joining of linear and circular plasmids results in linear fusion plasmids. The only apparent exception to this is the linear fusion plasmid lp32−6 + 10 in *B. garinii* PKi and PLa which is the result of a fusion of two circular plasmids cp32−6 and cp32−10, but the presence of telomeres (indicative of linear plasmids) on this plasmid suggests that an additional fusion event(s) occurred that contributed the telomeres (Fig 1 and S2 Fig, S1 Table).

Our analysis shows that 105 (9.8%) of the 1076 panel plasmids are “fusion plasmids” that carry multiple PFam32 genes. This is slightly higher than the 7.2% found by Akther et al. 2024 [72] using a smaller set of isolates; but we identified additional fusion plasmids that the aforementioned study failed to recognize (S3 Table). Fusions of multiple linear (n = 14) as well as multiple circular (n = 45) plasmids are present as well as fusions between linear and circular (n = 46) plasmids. The fusion plasmids are included in the previously mentioned linear and circular plasmid numbers and in the whole data set range from 0 to 4 per isolate (m = 1.0, Q1 = 0.0, Q3 = 2.0).

The most abundant plasmid types are as follows: Plasmids lp54 and cp26 are the only plasmid types found in all 86 genomes, followed by lp17 in 84 genomes (missing in *B. carolinensis* SCW-22 and *B. garinii* PMa) (Fig 2B and 2C). The lp36 type PFam32 gene is present in 76 genomes (88%) – as non-fused plasmid in 72 genomes (84%) with one PFam32 gene and as fusion plasmid in 4 genomes (5%) (Fig 2B, S1 Table); lp28−3 PFam32 type is present in 63 genomes (73%) – as non-fused plasmid in 57 genomes (66%) and as fusion plasmid in 6 genomes (7%); lp25 PFam32 type is present in 63 genomes (73%) – as non-fused plasmid in 41 genomes (48%) and as fusion plasmid in 22 genomes (26%); cp32−5 PFam32 type is present in 63 genomes (73%) – as non-fused plasmid in 51 genomes (59%) and as fusion plasmid in 12 genomes (14%); lp28−4 PFam32 type is present in 58 genomes (67%) – as non-fused plasmid in 39 genomes (45%) and as fusion plasmid in 19 genomes (22%). The remaining less abundant PFam32 types are each present in less than 55 genomes including non-fused and fusion plasmid forms. Several plasmid types (mainly fusion plasmids) are only found in single genomes (Fig 2C). Additionally, plasmid co-occurrences were analyzed and results are presented in S1 File.

### Do plasmid numbers and compatibility types correlate with natural history features?

#### Plasmid variation among *B. burgdorferi* s.l. species and populations.

**Fig 3:**
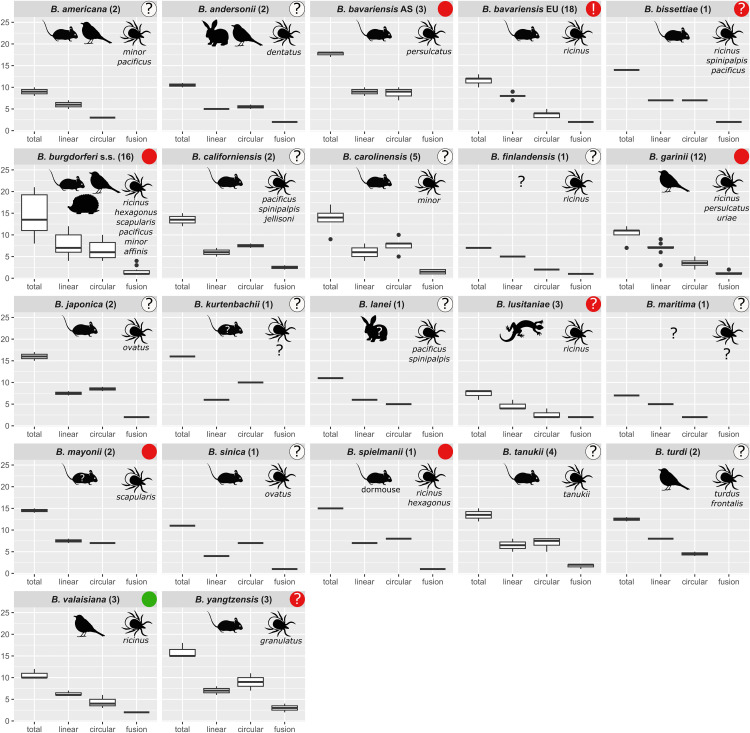
Boxplots per species/populations showing the total, linear, circular and fusion plasmid number ranges. For the species *B. bavariensis* two separate boxplots for the Asian and European population are shown. The category total contains linear, circular and fusion plasmids, while linear and circular plasmids contain fusion plasmids. Boxplot headers include information about species/populations and the number of isolates in brackets. Reservoir hosts are shown as icons according to Fig 1 (unknown hosts are indicated by “?”; for insectivores and carnivores a hedgehog icon is used). Tick vector species are listed. Pathogenicity information is shown in the right upper corner in different colors (green: nonpathogenic, red: pathogenic, red and”?”: potentially pathogenic, red and “!”: highly invasive, white and”?”: unknown).

Plasmid number ranges for each of the 21 species are shown in the Fig 3 boxplots with additional information included on reservoir host, vector association and pathogenicity; for the species *B. bavariensis* two separate boxplots for the Asian and European population are shown.

**Fig 4:**
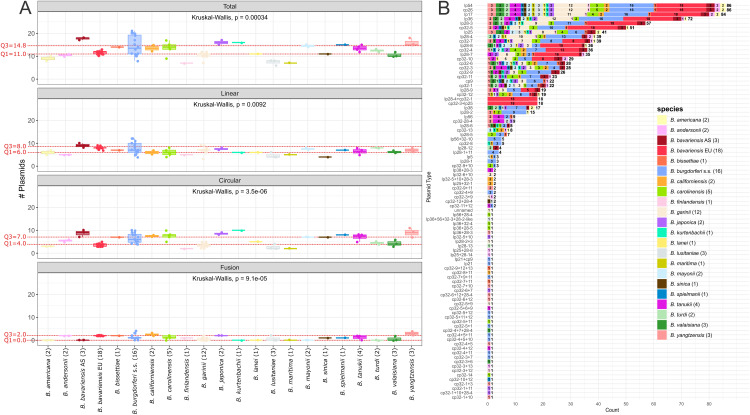
Species specific analyses of plasmid numbers per topology and plasmid types. **(A)** Boxplot showing the spread of plasmid numbers per topology grouped by species. Colors correspond to species (legend is shown in **B**). The category total contains linear, circular and fusion plasmids, while linear and circular plasmids contain fusion plasmids. Kruskal-Wallis results are shown in the boxplots. For comparison, the first quartile (Q1_overall_) and third quartile (Q3_overall_) of the overall plasmid topology distribution (calculated across all samples) are indicated in the species-grouped boxplot as red dashed lines. These reference quartiles do not represent the quartiles of the species groups themselves but serve to identify species whose median plasmid numbers exceeds the upper quartile (Q3_overall_) or falls below the lower quartile (Q1_overall_) of the overall plasmid number distribution. **(B)** Barplot showing the prevalence of plasmid types per species including the actual fusion plasmids. The length of the bars corresponds to the count of the plasmid type in the total dataset and the exact number is shown at the right tip of the bar. Bars are colored according to the prevalence per species and corresponding counts are shown as numbers in the colored bar.

In addition, for each plasmid topology (total, linear, circular and fusion) plasmid number ranges grouped by species are shown in Fig 4A. The precise values used to generate the boxplots are listed in S5 Table.

The number of plasmid types per isolate varies substantially within and among species (total, linear, circular, and fusion plasmids, Kruskal-Wallis: *p* < 0.05) (Fig 4A, S5 Table). To identify species that deviate from the “normal” plasmid numbers observed in the overall dataset (between Q1_overall_ and Q3_overall_), we compared the median plasmid number of each species (m_species_) with the quartiles of the overall plasmid topology boxplot (Q1_overall_ and Q3_overall_, Fig 2A). If the species median is higher than Q3_overall_, this means that the species has an unusual high plasmid number. Likewise, if the species median is lower than Q1_overall_, this means that the species has an unusual low plasmid number.

**Total plasmid numbers.** A noticeably high number of total plasmids (m_species_total_ > Q3_overall_total_ = 14.8) is present in *B. spielmanii* (m = 15.0), *B. yangtzensis* (m = 15.0), *B. kurtenbachii* (m = 16.0), *B. japonica* (m = 16.0) and the Asian *B. bavariensis* population (m = 18.0). All these species are associated with rodents-only as reservoir hosts (probable host of *B. kurtenbachii*). In contrast, lower total plasmid numbers (m_species_total_ < Q1_overall_total_ = 11.0) are observed in *B. andersonii* (m = 10.5), *B. valaisiana* (m = 10.0), *B. americana* (m = 9.0), *B. lusitaniae* (m = 8.0), *B. maritima* (m = 7.0), and *B. finlandensis* (m = 7.0). No common reservoir hosts and tick vectors appear to exist for these species, but we noticed that they are not adapted to rodents-only. *Borrelia burgdorferi* s.s. has a median of 13.5 total plasmids and therefore did not have a noticeably high or low mean plasmid number but showed the widest range of plasmid numbers (see below).

**Linear plasmid numbers.** Linear plasmid numbers have a lower variability than circular ones and only the Asian *B. bavariensis* population with a median of 9.0 linear plasmids has numbers higher than the Q3_overall_linear_ = 8.0 value of the whole dataset. *Borrelia andersonii*, *B. finlandensis*, *B. maritima*, *B. sinica*, and *B. lusitaniae* have noticeably low linear plasmid numbers with median values of 4.0–5.0 and medians lower than Q1_overall_linear_ = 6.0.

**Circular plasmid numbers.** The highest variation among species is observed in the circular plasmid numbers which are dominated by the cp32 type plasmids. Species with high median circular plasmid numbers (> Q3_overall_circular_ = 7.0) are the rodent-only associated species *B. californiensis*, *B. tanukii*, *B. carolinensis*, *B. spielmanii*, *B. japonica*, *B. yangtzensis*, Asian *B. bavariensis*, and *B. kurtenbachii* (median range from 7.5 to 10.0). Species with a low median number of circular plasmids (< Q1_overall_circular_ = 4.0) are *B. garinii*, *B. americana*, *B. finlandensis, B. maritima*, and *B. lusitaniae* (median range from 2.0 to 3.5).

**Fusion plasmid numbers.** Fusion plasmids are found in 15 species/populations (*B. andersonii*, European *B. bavariensis*, *B. bissettiae*, *B. burgdorferi* s.s., *B. californiensis*, *B. carolinensis*, *B. finlandensis*, *B. garinii*, *B. japonica*, *B. lusitaniae*, *B. sinica*, *B. spielmanii*, *B. tanukii*, *B. valaisiana*, and *B. yangtzensis*), with a maximum median of 3.0 in *B. yangtzensis*. The remaining 7 species/populations (*B. americana*, Asian *B. bavariensis*, *B. kurtenbachii*, *B. lanei*, *B. maritima*, *B. mayonii*, *B. turdi*) do not contain fusion plasmids, but for several of these only one genome has been sequenced.

**Plasmid number ranges.** The widest interquartile range (IQR = Q3-Q1) of total, linear, and circular plasmid numbers are observed in *B. burgdorferi* s.s. (IQR_total_ = 8.3, IQR_linear_ = 4.0, IQR_circular_ = 3.5, respectively), while *B. tanukii* has the maximal IQR with regard to fusion plasmid numbers (IQR_fusion_ = 1.3). While for a majority of species (n = 18) of the dataset a low number of isolates (≤5) and their genomes are included, *B. garinii* (n = 12), *B. burgdorferi* s.s. (n = 16), and the European *B. bavariensis* (n = 18) are represented by substantially more isolates. The high number of *B. burgdorferi* s.s. genomes may explain its observed wide IQR (IQR_total_ = 8.3) in plasmid numbers, while the generalist nature of this species with multiple reservoir hosts and multiple vectors may also be a reason. In contrast to *B. burgdorferi* s.s., the IQR of plasmid numbers in the 12 *B. garinii* genomes is narrow (IQR_total_ = 1.0). This species is associated with a broad range of tick species, while only birds (of several different taxa, e.g., passerine birds and seabirds) serve as reservoir hosts. Although the European population of *B. bavariensis* has the highest number of analyzed genomes, it has a very narrow range of plasmid numbers with all strains carrying 10–13 plasmids (IQR_total_ = 1.0). In the following section, the two *B. bavariensis* populations are compared in more detail.

**Comparison of plasmid numbers of the Asian *vs* European *B. bavariensis* population.** The species *B. bavariensis* has two populations, a heterogeneous Asian and a homogeneous European population [6,24,33]. It was previously found that the Asian population has significantly higher total plasmid numbers compared to the European isolates [33,113]. Examination of the plasmid numbers of these two populations on our larger data set (Figs 3 and 4A) indicates higher total and circular plasmid numbers in the Asian population than in the European populations, but these differences are not statistically significant. While there are comparable numbers of linear plasmids in both populations, fusion plasmids are only observed in the European population. Both populations have a narrow IQR range of plasmid numbers with (European isolates IQR_total_ = 1.0 and Asian isolates IQR_total_ = 0.5). The narrow IQR of the Asian isolates may be due to the limited number of three analyzed genomes but the European isolates have the highest number of analyzed genomes and also have a narrow IQR. While the Asian *B. bavariensis* population that is associated with *I. persulcatus* has extensive genetic variation implicating that the origin of the species was relative long ago. In contrast, the European population that is associated with *I. ricinus* is genetically very homogenous [6,24,33] and it has been suggested that the European population invaded Europe after a recent vector switch from *I. persulcatus* to *I. ricinus* leading to a genetic bottleneck. This idea is consistent with the presence of identical fusion plasmids in all European *B. bavariensis* isolates, while in all other cases fusion plasmids are present in only a few (≤ 5) isolates (see next section).

**Plasmid types.** To determine whether species- or population-specific plasmid types are present in the dataset, a barplot showing the prevalence of the actual plasmid types was constructed (with fusion plasmids as separate types) and is colored according to the *B. burgdorferi* s.l. species/populations (Fig 4B). Many plasmids (especially fusion plasmids) are only found in one or two genomes and thus often in only one species. This likely reflects the fact that the plasmid rearrangement process is ongoing, and these fusions have formed recently. In the remaining plasmids only five plasmids are limited to a single species/population. The linear fusion plasmids lp28−4 + cp32−1 and cp32−3 + lp25 are exclusively found in the 18 European *B. bavariensis* genomes (see note “a” in S1 Table). Plasmids lp28−1, lp28−1 + 11, and lp56 + 32−10 are only found in a subset of the *B. burgdorferi* s.s. genomes (three, four, and five out of 16, respectively). Nearly all plasmid types are found in multiple species, and no further convincing species- or population specific plasmids were detected.

**Comparison of the plasmid repertoire of species with high plasmid numbers *vs* species with low plasmid numbers.** To determine whether plasmid types occur more frequently in species with high plasmid numbers (m > 14.8) compared to low plasmid numbers (m < 11.0), we compared the plasmid repertoire of these groups. We hypothesize that plasmids that are only found in high plasmid number species and are not detected in the low plasmid number species may be dispensable in niche adaptation circumstances.

Ten isolates belonging to the species Asian *B. bavariensis, B. japonica, B. kurtenbachii*, *B. spielmanii*, and *B. yangtzensis* are in the high plasmid number category and were compared to 12 isolates belonging to the species *B. americana*, *B. andersonii*, *B. finlandensis, B. lusitaniae*, *B. maritima*, and *B. valaisiana* which are in the low plasmid number category (Table 4). The presence of PFam32 gene types was compared instead of plasmid numbers, as multiple PFam32 genes are present on one fusion plasmid.

**Table 4:** Comparison of the plasmid repertoire based on PFam32 loci of high plasmid number species (m_species_ > 14.8, 10 isolates) *vs* low plasmid number species (m_species_ < 11.0, 12 isolates). Only PFam32 loci that are present in at least one of these isolates are shown. Plasmid/ PFam32 presence is indicated green (complete plasmid) and red (incomplete plasmid), plasmid/ PFam32 absence is indicated in white. Additionally, fusion plasmids “X” and cp32-28-4 “$” (circular plasmid with syntenic gene contents to cp32 plasmid family but encodes a lp28−4 type PFam32 protein) are marked. The number of found PFam32 is shown per group as well as the number of PFam32 of lp28 and cp32 plasmids.

								lp28s													cp32s											
Group	Isolate	Species	# plasmids	lp54	cp26	lp17	lp25	lp28−2	lp28−3	lp28−4	lp28−6	lp28−7	lp28−8	lp28−9	lp28−12	lp28−14	lp36	lp38	lp56	cp9	cp32−1	cp32−3	cp32−4	cp32−5	cp32−6	cp32−7	cp32−8	cp32−9	cp32−10	cp32−11	cp32−12	cp32−13
low plasmid number species	SCW-30h	*B. americana*	8																													
	SCW-41	*B. americana*	10																													
	21038	*B. andersonii*	10				X						X									X						X				
	MOD-5	*B. andersonii*	11				X									X						X										X
	Z11	*B. finlandensis*	7																			X									X	
	PoHL1	*B. lusitaniae*	6							$																						
	PotiB3	*B. lusitaniae*	8							$																						
	PotiB2	*B. lusitaniae*	8					X		X$							X		X			X									X	
	CA690	*B. maritima*	8																													
	89B13	*B. valaisiana*	10																													
	100B40	*B. valaisiana*	10																													
	Am501	*B. valaisiana*	12							X$												X	X		X	X						
	∑ PFam32			12	12	12	11	3	2	9	1	1	7	0	0	1	7	5	1	3	3	6	1	1	3	4	0	2	2	2	5	3
	% PFam32			100	100	100	92	25	17	75	8	8	58	0	0	8	58	42	8	25	25	50	8	8	25	33	0	17	17	17	42	25
	∑ PFam32 of lp28s and cp32s							24													32											
	∅ ∑ PFam32 of lp28s and cp32s per isolate							2													3											
high plasmid number species	Prm7564−11	Asian *B. bavariensis*	17																													
	Tmsk976–2013	Asian *B. bavariensis*	18																													
	NT24	Asian *B. bavariensis*	18																													
	Miyazaki2E	*B. japonica*	15							X$											X				X	X			X			
	HO14	*B. japonica*	17							$											X			X					X	X		
	25015	*B. kurtenbachii*	16																													
	PMew	*B. spielmanii*	15																										X		X	
	Miyako4E	*B. yangtzensis*	15						X	$							X									X			X			
	Okinawa-CW62	*B. yangtzensis*	15							X$											X		X	X	X			X	X	X	X	
	Okinawa-CW61	*B. yangtzensis*	18							$											X	X			X			X		(X)	X	
	∑ PFam32			10	10	10	8	2	8	8	3	3	4	5	2	0	10	3	2	5	7	9	9	9	9	8	1	9	9	8	7	1
	% PFam32			100	100	100	80	20	80	80	30	30	40	50	20	0	100	30	20	50	70	90	90	90	90	80	10	90	90	80	70	10
	∑ PFam32 of lp28s and cp32s							35													86											
	∅ ∑ PFam32 of lp28s and cp32s per isolate							4													9											

The PFam32 loci of lp54, cp26, lp17, and lp36 are present in all isolates of the high plasmid number species. While lp54, cp26, and lp17 are also present in all isolates of the low plasmid number species, lp36 PFam32 type genes are only present in 58% of these isolates. We also compared the presence of lp28s and cp32s PFam32 types in these two groups. A lower number of lp28 and even more clearly of cp32 PFam32 types are present in species with low plasmid counts. On average four lp28 PFam32 types are found per isolate in species with high plasmid numbers, while it is only two per isolate of species with low plasmid numbers. The PFam32 type lp28−4 occur most frequently in both groups (present in 80% and 75% of isolates with high and low plasmid number, respectively). Also, lp28−8 plasmids are present in 40% and 58% of the isolates with high and low plasmid number counts, respectively. Particularly striking is lp28−3 PFam32 plasmids which are present in 80% of isolates belonging to species with high plasmid counts, but only in 17% of isolates of species with low plasmid counts; additionally, lp28−9 plasmids are present in 50% of isolates belonging to species with high plasmid counts but are completely missing in isolates belonging to species with low plasmid counts. This distribution is even more noticeable in the case of cp32 PFam32 types: isolates of species with high plasmid numbers have on average nine cp32 PFam32 types, while only three are present in isolates of species with low plasmid numbers. An exception is cp32−13 with 10% to 25% presence in isolates belonging to species with high *vs* low plasmid counts, respectively. The remaining cp32 plasmids clearly decreased with decreasing total plasmid number. For example, cp32−4 and cp32−5 were found in 90% of the isolates of species with high plasmid numbers, but only in 8% of isolates of species with low plasmid numbers. Presently, we can only speculate that this may show that depending on the ecological niche, it can be less important for *B. burgdorferi* s.l. to have numerous lp28 and/or cp32 plasmids. This suggestion is supported by the recent findings that *B. burgdorferi* s.s. B31 lacking cp32 plasmids is fully infective in mice [114]. Nevertheless, we noticed that all our analyzed isolates have cp32 plasmids, except for *B. lusitaniae* PoHL-1 carrying only a cp32-28-4 that has syntenic gene contents with the cp32 plasmids but encode a lp28−4 type PFam32 protein [72].

### Plasmid variation among different ecological niche adaptations (reservoir host and vector) and pathogenicity groups

The ecological niche adaptations and pathogenicity potential of the 86 *B. burgdorferi* s.l. isolates analyzed here are as follows (Fig 1, S1 Table for details):

**Reservoir host adaptation:** Isolates are adapted to birds-only (n = 17); rodents-only (n = 40); rodents and birds (n = 2); rodents, birds, insectivores, and carnivores (n = 16); lizards (n = 3), lagomorphs and birds (n = 2); potentially rodents (rodents?) (n = 3), potentially lagomorphs (lagomorphs?) (n = 1), unknown (n = 2). Accordingly, isolates are adapted to a single host (n = 60), multiple hosts (n = 20), potentially a single host (single?) (n = 4), and unknown hosts (n = 2). Although birds and rodents are large groups that include many genera and species, we use the coarse divisions to examine whether any plasmid patterns are present.

**Vector adaptation:** The *B. burgdorferi* s.l. species under consideration here are currently known to be effectively transmitted by one or more of the following sixteen *Ixodes* tick species as vector: *I. affinis, I. dentatus, I. frontalis, I. granulatus, I. hexagonus, I. jellisoni, I. minor, I. ovatus, I. pacificus, I. persulcatus, I. ricinus, I. scapularis, I. spinipalpis, I. tanukii, I. turdus, I. uriae*. For example, *B. burgdorferi* s.s. is vectored by *I. ricinus*, *I. hexagonus*, *I. scapularis*, *I. pacificus*, *I. minor* and *I. affinis*, while *B. valaisiana* is only vectored by *I. ricinus*. From a species perspective *B. bavariensis* is vectored by *I. ricinus* and *I. persulcatus*, while considering populations European *B. bavariensis* have only been associated with *I. ricinus* and the Asian *B. bavariensis* population is solely associated with *I. persulcatus* [17,22,23]. Thus, each of these two populations is considered to be associated with a single vector species. Accordingly, isolates are vectored by a single tick species (n = 47), multiple (n = 37), or an unknown tick vector (n = 2).

**Human pathogenicity:** The 86 isolates can be grouped by pathogenicity status of their species (pathogenic: causes human disease, potentially pathogenic: rarely isolated from humans but not yet clearly associated with disease, nonpathogenic: not associated with human disease, unknown: not isolated from humans, but vectored by ticks that are not known to bite humans at any frequency). The majority of isolates (n = 52) belong to the five human pathogenic species *B. bavariensis*, *B. burgdorferi* s.s., *B. garinii*, *B. mayonii*, and *B. spielmanii.* The species *B. bissettiae*, *B. lusitaniae*, *B. yangtzensis* were categorized as potentially human pathogenic isolates (n = 7) as they have less frequently been reported to infect humans [7–14]. Please note that in the case report describing a *B. yangtzensis* infection in Japan, the species was incorrectly named *B. valaisiana* because the closely related species *B. yangtzensis* was not described at that time [12–14]. Isolates that actually belong to the species *B. valaisiana* (n = 3) were considered nonpathogenic for humans for the following reasons: While *B. valaisiana* is commonly found in questing *I. ricinus* ticks (a human-biting tick) in European regions, regular infections caused by *B. valaisiana* would be expected. However, to date not a single isolate has been obtained from a human infection (only DNA evidence was provided in a few records) [15]. The pathogenicity status of the remaining 12 species (n = 24 isolates) is unknown, because, at this point, they have not been isolated from humans (*B. americana*, *B. andersonii*, *B. californiensis*, *B. carolinensis*, *B. finlandensis*, *B. japonica*, *B. kurtenbachii*, *B. lanei*, *B. maritima*, *B. sinica*, *B. tanukii*, *B. turdi*).

The isolates were grouped according to reservoir host adaptation, vector adaptation, and human pathogenicity potential and these groups were searched for trends in plasmid numbers and types. Detailed results are shown in S1 File and S3 Fig (various host classes), S4 Fig (single *vs* multiple hosts), S5 Fig (various vector classes), S6 Fig (single *vs* multiple vectors), and S7 Fig (pathogenicity). In addition, plasmid co-occurrences were analyzed (S1 File, S8 Fig). Major results are summarized in Table 5.

**Table 5:** Major results of plasmid analyses among different ecological niche adaptation (reservoir host and vector) and pathogenicity. Detailed results are described in supplementary information (S1 File and S3 – S8 Figs).

Category	Reservoir host	Vector	Pathogenicity
**Plasmid numbers**	**various host classes:** • rodents-only *vs* birds-only: significantly higher total, circular and fusion plasmid numbers in rodents-only • rodents-only: trend of more circular plasmids relative to linear plasmids • birds-only: trend of more linear plasmids relative to circular plasmids	**various vector species:** • significant differences in all plasmid topologies comparing the different vector species	• mostly no significant differences • trend of higher total and linear plasmid numbers in pathogenic *vs* nonpathogenic
	**single *vs* multiple hosts:** • no significant differences comparing single *vs* multiple hosts • higher IQR for total and linear plasmids numbers in multiple hosts *vs* single	**single *vs* multiple vectors:** • mostly no significant differences comparing single *vs* multiple vectors • no differences in IQR	
**Plasmid types**	• exclusively in EU *B. bavariensis* (“rodent-only”, single host): lp28−4 + cp32−1 cp32−3 + lp25 • exclusively in a subset of *B. burgdorferi* s.s. (“rodents, birds, insectivores, and carnivores”, multiple hosts): lp56 + 32−10 lp28−1 + 11 lp28−1 • exclusively found in single host isolates: cp32-28-4^$^, lp28−12 • cp32−5 and cp32−4 more often in rodents-only *vs* birds-only	• exclusively in EU *B. bavariensis* (“*I. ricinus*”, single vector): lp28−4 + cp32−1 cp32−3 + lp25 • exclusively in a subset of *B. burgdorferi* s.s. (“*I. ricinus, I. hexagonus, I. scapularis, I. pacificus, I. minor, I. affinis*”, multiple vectors): lp56 + 32−10 lp28−1 + 11 lp28−1 • exclusively found in single vector isolates: cp32-28-4^$^	• exclusively found in pathogenic isolates (EU *B. bavariensis* or subset of *B. burgdorferi* s.s.): lp28−4 + cp32−1 cp32−3 + lp25 lp56 + 32−10 lp28−1 + 11 lp28−1 • cp32−5 is frequently present in pathogenic isolates, but is absent in nonpathogenic isolates
**Plasmid** **co-occurrence**	• higher percentage of plasmid correlations in single *vs* multiple hosts • higher percentage of plasmid correlations in rodents-only *vs* birds-only	• higher percentage of plasmid correlations in single *vs* multiple vectors	• noticeable increased co-occurrences with other plasmids for each lp25, lp28−4, and lp38 in pathogenic isolates *vs* all isolates

$ cp32-28-4: plasmid has syntenic gene content with the cp32 plasmid family; however, it encodes a lp28−4 type PFam32 protein

## Discussion

In this study, we analyzed the genome composition of the currently available high-quality *Borrelia burgdorferi* s.l. genomes (86 genomes of 21 species). Among these, we analyzed the performance of the high-fidelity approach for *B. burgdorferi* s.l. genome assembly [73] with 30 newly sequenced isolates and assessed their completeness levels. We paid particular attention to the plasmid location of the *vls/vlsE* as a particularly challenging locus to assemble. On the other hand, plasmid composition (presence/absence, number and plasmid types) was analyzed with the aim to identify if some plasmids may be involved in ecological adaptation or human pathogenicity. For this, the plasmids in our dataset were grouped by species, vertebrate reservoir host and tick vector adaptation, as well as human pathogenicity potential.

### The high-fidelity assembly approach and the challenging *vls/vlsE* locus

During assessment of the completeness of the newly sequenced genomes, we confirmed that the comparison and combination of multiple assemblies, generated by different software, are essential steps to obtain best genome reconstruction results. Of the different assemblers used, HiCanu and MGA assemblers performed best for the genome reconstruction. Incompletely reconstructed genomes were due to the following reasons: (i) using PacBio RS II instrument data that cannot utilize improved assemblers such as MGA, IPA, or HiCanu and therefore only HGAP assemblies are available, (ii) DNA of limited quantity and/or quality, (iii) difficulty in properly reconstructing the lp28s and cp32s plasmids or the *vls/vlsE* carrying plasmid. For some newly sequenced isolates, the *vls/vlsE* locus was only found in Illumina SPAdes contigs and not in the PacBio assembly or the *vls/vlsE* was completely missing. Potential explanations for incomplete or missing *vls/vlsE* may include (i) that the plasmid was in the process of being lost as a result of *in vitro* cultivation and so had low copy number per cell [115–120], (ii) that this part of the plasmid was lost during library preparation, (iii) that the sequencing or assembly process failed or (iv) that the *vls/vlsE* locus was not present in the isolate. Some studies report that the *vls/vlsE* is essential for *B. burgdorferi* s.s. to survive and persist in the host [42,43,57,59,111]. However, we found that the genome assemblies of some isolates of several species (European *B. bavariensis*, *B. burgdorferi* s.s., *B. carolinensis*, *B. garinii*, *B. tanukii*, *B. valaisiana*) or even all isolates analyzed in *B. californiensis*, *B. sinica*, and *B. yangtzensis* completely lacked this locus. For some genomes with missing *vls/vlsE* locus the raw reads were available and were searched for *vls/vlsE*. These analyses revealed that for two *B. yangtzensis* strains (Miyako4E and Okinawa-CW2) *vls/vlsE* sequences were present in the read data, showing that the locus was actually present in the genome but failed to be properly reconstructed during genome assembly. While for several further isolates no reads or only few reads matched to the *vls/vlsE* reference, supporting the suggestion that the *vls/vlsE* locus is missing in the genome. It remains ambiguous whether the missing of *vls/vlsE* locus in assembled genomes is a sequencing/assembly artifact and whether the plasmid/locus was present in the wild type. In addition, it is currently unclear whether the *vls/vlsE* is also essential in species other than *B. burgdorferi* s.s. and consequently the question arises what could replace it to ensure persistence in the host.

### The location of *vls/vlsE* system underlines the plasticity of *B. burgdorferi* s.l. genomes

In the analyzed dataset, the *vls/vlsE* system in various species was located on plasmids lp28−1, lp28−1 + 11, lp28−3, lp28−8, lp25 + 28−8, lp25, lp36 and lp32−7. In addition, Casjens et al. 2017 [30] and Casjens et al. 2018 [31] reported *vls/vlsE* on lp28−9, lp32−3 and lp32−6 plasmids in *B. burgdorferi* s.l. isolates that are not in the current study. Combined, these analyses have found *vls/vlsE* in the genome assemblies of the following 19 *B. burgdorferi* s.l. species: *B. afzelii, B. americana, B. andersonii, B. bavariensis* (European and Asian population), *B. bissettiae, B. burgdorferi* s.s., *B. carolinensis, B. finlandensis*, *B. garinii*, *B. japonica, B. kurtenbachii, B. lanei, B. lusitaniae, B. maritima*, *B. mayonii*, *B. spielmanii, B. tanukii, B. turdi*, *B. valaisiana* and on ten different PFam32 types. These findings strikingly underpin the dynamic nature of the *B. burgdorferi* s.l. plasmids.

### Genome composition covers all known plasmid types

The 86 genomes contain 35 different PFam32 plasmid types, including the PFam32 carrying cp9, and additionally cp9 and lp5 plasmids that lack the PFam32 gene but carry the PFam57 gene. There was an additional linear plasmid in one of the newly sequenced isolates that is lacking the PFam32 gene but encodes PFam57 and was called “unnamed”. Akther et al. 2024 [72] proposed to redefine the “lp5” group and to include linear plasmids that are lacking the PFam32 gene but present the PFam57 genes, despite the fact that these plasmids have no gene content other than lacking PFam32 and presenting PFam57 gene in common. Further analyses on PFam57 genes are needed to resolve the issue. No new plasmid PFam32 types were found in the 30 new genomes presented here suggesting that if any additional undiscovered plasmid compatibility types exist in nature, they are rare or are present in species or geographical regions that have not been studied extensively. The dataset of 86 genomes analyzed in our study contained all known plasmid compatibility types described to date, making it a good basis for conducting comparative plasmid analyses. Plasmids lp54, cp26 and lp17 are universally present in *B. burgdorferi* s.l. isolates [28,32], but lp17 was missing in two of the analyzed isolates, and we speculate that this plasmid may have been lost during culture.

It is well known that plasmids can be lost during growth in culture [115–120] but the reasons are not fully understood. One possible reason for plasmid loss could be the cell cycle and segmentation mechanism in *B. burgdorferi* s.l.. The genome is replicated continuously, instead of clearly separating the replication and division phases [121]. This leads to regularly spaced multiple genome copies in one elongated *B. burgdorferi* s.l. cell, but unequal copy numbers for each replicon [121]. The plasmid to chromosome ratio ranged from 0.5 to 1.4 for *B. burgdorferi* s.s. [121], slightly lower than previous reports that measured ranges from 1 to 2 or 3 [32,122,123]. Further studies reported even higher numbers of specific plasmid types: lp28−6 copies were approximately 10-fold higher compared to the other plasmids in *B. burgdorferi* s.s. JD1 [28] and up to five copies per chromosome were reported for lp17 in *B. bavariensis* [33]. In the newly sequenced *B. bavariensis*, *B. garinii* and *B. valaisiana* genomes we also found higher copies of lp17 (in average 9 copies per chromosome). The spacing and segmentation of the chromosome is controlled by two separate partitioning systems (ParA/ParZ and ParB/Smc) that ensure proper chromosome partitioning, while the plasmids are thought to use the ParA/ParB system (including members of the PFam32, PFam49, PFam50 and PFam57/62 gene cluster) [121,124]. Some of the linear *B. burgdorferi* s.s. plasmids (lp17, lp21, lp25 and lp28−3) showed high frequency of contact in the Hi-C maps suggesting interaction with the chromosome, while lower interaction was observed for plasmids cp32−3, cp32−7 and cp32−9 [124]. These authors speculated that these linear plasmids may “piggyback” on the chromosome to ensure their own segmentation and maintenance. Interestingly, the universally present plasmids lp54 and cp26 showed no discernible interaction with the chromosome, while interaction was reported for lp17 (absent in two of the 86 genomes analyzed here). However, it is still not known if the chromosome-plasmid interactions contribute to plasmid maintenance.

Our data corroborate previous studies [72,74] reporting that apart from the universally present plasmids lp54 and cp26 the most prevalent PFam32 types in *B. burgdorferi* s.l. are lp17, lp36, lp28−3, lp25, cp32−5 and lp28−4 (present in ≥ 67% of the analyzed genomes). These mainly correspond to those that are essential for *B. burgdorferi* s.s. B31 invasiveness in mice (cp26, lp54, lp25, lp28−1 and lp36) [36,42–50] and for tick gut colonization (lp25 and lp28−4) or tick-to-host transmission in *B. burgdorferi* s.s. (lp28−1) [47,48,50,51]. An exception is lp28−1 (separated into lp28−1 and lp28−1 + 11 types in our analysis) that is only found in 8% of our analyzed genomes, corresponding exclusively to a subset of the analyzed *B. burgdorferi* s.s. isolates. In the absence of experimental evidence, one could hypothesize that across the *B. burgdorferi* s.l. species common plasmid types cp26, lp54, lp25, lp36, and lp28−4 are important for reservoir host and tick vector interaction in species other than *B. burgdorferi* s.s. and that the plasmid lp28−1 may be exclusively associated with the species *B. burgdorferi* s.s., but this requires further investigation on the genes that are present.

Our results showed that 9.8% of the plasmids are the results of fusion of at least two plasmid PFam32 types. The function of fusion plasmids in *B. burgdorferi* s.l. is currently unknown. Fusion plasmids are known to occur also in other bacterial species and lead to increases in suitable cell range and contribute to stability [125,126], and can add expanded genetic content that may enhance pathogenic potential [127]. An example for the latter, are fusion plasmids in *Klebsiella pneumoniae* that contain antimicrobial resistance and hyper-virulence determinants [127]. The functional role of fusion plasmids in *B. burgdorferi* s.l. requires more detailed analyses of the genes they carry.

In the past, fusion plasmids have not always been named according to all present PFam32 types. For example, lp28−1 of B31 has two PFam32 types, one of lp28−1 and one of lp28−11 and here we named it lp28−1 + 11. Similarly, lp56 of B31 carries PFam32 types of lp56 and cp32−10 and is named lp56 + 32−10 in this study (S3 Table). We also recognized that plasmids sometimes carry additional PFam32 pseudogenes. While this suggests fusion of plasmids in the past, it raises the question of whether plasmids presenting short additional PFam32 fragments should also be considered as fusion plasmids and whether they ought to be taken into account for naming purposes. In this study, intact PFam32 and pseudogenes were considered but stringent thresholds (≥ 86% nucleotide identity and ≥ 90% coverage) were applied both for determining them as fusion plasmids and for nomenclature. We did this to ensure that the fusion plasmids were accurately detected and assigned. Short PFam32 fragments may pose a risk of misclassification, particularly regarding sequence similarity within the cp32 plasmids and within the lp28 plasmids and were therefore excluded. Furthermore, we are aware that, besides the presence of multiple PFam32 genes, there are additional indications suggesting that the plasmid is actually a fusion plasmid: (i) if a linear plasmid (identified by the presence of telomeres) carries one or multiple PFam32 genes typically found on circular plasmids, the plasmid is probably not only the result of fused circular plasmids as a linear plasmid contributed the telomeres (e.g., lp32−6 + 10 found in *B. garinii* PKi and PLa), (ii) if a non-cp32 plasmid (i.e., presents a non-cp32 PFam32 gene), but carries genes that are only found on cp32 plasmids, such as *erp* gene family or phage-like genes, this also likely indicates a past fusion event. As a result, based on the search for multiple PFam32 genes and using stringent settings, only a minimal but confidently assigned set of fusion plasmids was identified. To obtain a clearer and more comprehensive picture of the existing fusion plasmids, further and more extensive analyses will be required.

### Are plasmid numbers and/or plasmid types associated with isolates of the same ecological adaptations or pathogenicity potential?

It is generally assumed that isolates of *B. burgdorferi* s.l. species occupy identical or very similar ecological niches [4,18,20]. This may not always be the case as it has been suggested that *B. burgdorferi* s.s. isolates differ slightly in the niches they occupy and their degrees of pathogenicity in humans [128,129]. It is also possible that the distinct populations of species, such as *B. lusitaniae* [130,131] or *B. bavariensis* [24], are the result of differing ecological niche adaptation. This is well described for *B. bavariensis* [17], where Asian *B. bavariensis* are associated with only *I. persulcatus* while the highly invasive European *B. bavariensis* are solely associated with *I. ricinus* [17,22,23]. We hypothesize that these differences should be reflected in the gene or plasmid repertoire of such populations.

The plasmid repertoire was highly variable even for isolates of the same species, with only isolates of the European *B. bavariensis* population showed reduced variability in plasmid content although the plasmid content in different isolates was not identical. Only a few species-/population-specific plasmids were found: (i) fusion plasmids lp28−4 + cp32−1 and cp32−3 + lp25 are exclusively found in all isolates of the European *B. bavariensis* population. (ii) lp28−1 is only found in a subset of *B. burgdorferi* s.s. (OspC types A, K and L of this species; determined in Akther et al. 2024 [72] and are presented in Fig 4B of their study). This observation supports the hypothesis put forward by Casjens et al. 2018 [31] that the plasmid compatibility types arose early (even before the split of *B. burgdorferi* s.l., relapsing fever and other groups) and with occasional species plasmid transfer, only a few (if any) species-specific plasmids types have emerged. These plasmids will be examined in more detail below.

The European *B. bavariensis* population is considered highly invasive in humans [17,21,24,33] and the linear fusion plasmids lp28−4 + cp32−1 and cp32−3 + lp25 are exclusively found in all analyzed isolates of that European population. As it is known that fusion plasmids in *Klebsiella pneumoniae* contain hyper-virulence determinants [127], one could speculate that these plasmids may contribute to the increased human invasiveness of the European *B. bavariensis* population. Other fusion plasmid types are limited to only a few isolates and are scattered apparently randomly across the various *B. burgdorferi* s.l. species and isolates (S2 Fig, S1 Table) suggesting that they may be randomly formed and transitory. Like previous studies using incomplete genomes based on Illumina short reads [33,113], we found (i) higher plasmid numbers in Asian *B. bavariensis* compared to the European ones, (ii) a rather stable plasmid repertoire in European *B. bavariensis*, perhaps due to a recent ecological adaptation (adaptation to the tick vector species *I. ricinus*) and population expansion of the surviving invaders resulting in a genetic bottleneck [17,24].

*Borrelia burgdorferi* s.s. isolates with OspC type A show a high degree of dissemination in humans, and this has been linked to the plasmids lp28−1 and lp56 [74]. In our study, lp28−1 and lp56 (sometimes occurring as fusion plasmid lp28−1 + 11 and lp56 + 32−10, respectively) were found in some but not all OspC type A isolates, and also in other OspC types. In addition, lp56 is also found in other species (15 genomes of the species *B. bissettiae*, *B. carolinensis*, *B. lusitaniae*, *B. tanukii*, *B. yangtzensis*), some of which are potentially human pathogenic (*B. bissettiae*, *B. lusitaniae*, *B. yangtzensis)*. These observations further underline *B. burgdorferi* s.l. plasmid dynamics even in different isolates/lineages of a single species and the need of further gene-based analyses.

Searching for trends in the plasmids associated with reservoir host our analyses revealed three key findings: i) all species with a strikingly high number of plasmids (median > 14.8) are associated (or are hypothesized to be associated) with rodents-only as reservoir hosts, ii) rodents-only associated isolates have significant higher numbers of total, circular and fusion plasmids as well as higher percentage of positive plasmid co-occurrences compared to the birds-only associated group, iii) considering the proportions within the whole dataset (86 genomes) grouped by plasmid topology, we found a significantly higher number of linear plasmids compared to circular plasmids which is in accordance with Casjens et al. 2018 [31]. While birds-only associated species have indeed a higher proportion of linear relative to circular plasmids, rodents-only associated isolates show the opposite pattern with more circular plasmids relative to linear ones (except for the European *B. bavariensis* isolates).

Genes encoded on linear plasmids are primarily responsible for the host-pathogen and vector-pathogen interaction, while circular plasmids encode only a few relevant outer surface proteins (such as *ospC* on cp26 or *erps* on cp32 plasmids) [32,41]. Linear plasmids are essential for invasiveness in mice in *B. burgdorferi* s.s. B31 (lp54, lp25, lp28−1 and lp36, but also the circular plasmid cp26) and are also important for tick gut colonization (lp25 and lp28−4) or tick-to-host transmission by salivary gland invasion (lp28−1) in *B. burgdorferi* s.s. [36,42–51]. Some important genes encoded on these linear plasmids in B31 are *ospA* (lp54), *vls/vlsE* (lp28−1), *dbpA* and *dbpB* (lp54), *bbk32* (lp36), CRASP genes (lp54, lp28−3) [32,41]. The observation of increased proportion of circular plasmids relative to linear ones in the rodents-associated group raises questions about which genes on these circular plasmids might play a role in host adaptation, especially regarding adaptation to rodents-only. However, addressing this will require further comparative gene analyses.

It has been suggested that particular plasmids might allow survival in specific reservoir hosts or vectors (e.g., seabird associated *B. garinii* contain fewer plasmids than isolates associated with terrestrial birds [100] or high cp32 variety in *B. burgdorferi* s.s. which has a wide range of hosts [38]). Thus, it might have been expected that species with a narrow reservoir host and/or vector range (single host class or vector species) would show lower plasmid numbers than species with multiple host classes or vector species. Perhaps surprisingly, we did not find significant differences in the plasmid numbers comparing isolates associated with single *vs* multiple reservoir hosts or vectors. Indeed, the specialized species *B. spielmanii* isolate PMew which is specialized to the dormouse (a single reservoir host) [19] contains an above average of 15 plasmids (whole dataset: m = 12.5, Q3 = 14.8). However, we recognized that more plasmids show co-occurrences with other plasmids in single *vs* multiple reservoir hosts and vectors which may be a result of specialization. We additionally observed higher IQR in isolates associated with multiple *vs* single host, while this was not true for the comparison of multiple *vs* single vector isolates. While it remains to be confirmed by more extensive analysis, this finding may suggest that the range of associated reservoir hosts may contribute to greater plasmid number diversity while the vector range does not influence it.

We noticed a slight trend of higher total and linear plasmid numbers in pathogenic than in nonpathogenic isolates, but the difference was not significant, and results should be interpreted with caution due to limited sample size of nonpathogenic isolates. We also observed a trend of some plasmids to occur more frequently in pathogenic isolates. The most noticeable is the non-fused plasmid cp32−5 as it was present in a high number of isolates (n = 51 in total, pathogenic n = 39, potentially pathogenic n = 3, unknown n = 9) and is absent in the three nonpathogenic isolates. We noticed that this plasmid also occurred more frequently in rodents-only adapted isolates compared to birds-only ones. Although this remains speculative, the plasmid cp32−5 may be an interesting candidate plasmid for future gene-based investigations seeking to gain improved insight into the molecular factors that are involved in ecological niche adaption and human pathogenicity.

## Conclusion and outlook

The number of *B. burgdorferi* s.l. complete genome sequences has grown considerably in the last several years, and we report the first search for possible correlations between plasmid numbers and plasmid types with *B. burgdorferi s.l.* species, vertebrate reservoir hosts, tick vectors, and human pathogenicity. Analyzing the completeness and plasmid repertoire of a wide range of *B. burgdorferi* s.l. genomes provide an excellent basis for comparative genomics. A limitation of the study is that for some *B. burgdorferi* s.l. species, populations or isolates knowledge on reservoir host or vector associations are not yet fully understood. A further limitation is that only a small number of isolates were available for some species and groups, which also led to uneven group sizes in some comparisons. Furthermore, it is well established that plasmids can be lost during cultivation [115–120] and therefore, we cannot ensure that the plasmid repertoire presented here is fully representative of the naturally occurring state. However, this study provides an important starting point for discovery of initial insights into the identification of candidate plasmids for further analyses.

The major findings of this study are (i) that the high-fidelity approach for *Borrelia* genome reconstruction [73] resulted in high-quality genomes, while incomplete genome reconstruction was mainly due to older sequencing instruments, limited DNA quality/quantity or complicated plasmids (lp28s, cp32s, *vls/vlsE* carrying plasmids); (ii) the variability of the prevalence and plasmid location of the *vls/vlsE*; (iii) the overall high variability of the plasmid repertoire and plasmid numbers even within species.

We conclude that *B. burgdorferi* s.l. plasmid repertoire is seemingly chaotic in numbers and presence/absence even within species – the wild, wild west of plasmids. Only few plasmids were identified that are associated with isolates with particular adaptations and even these were usually not absolute correlations. Our analysis shows that ecological adaptations and human pathogenicity potential of *B. burgdorferi* s.l. isolates are not linked to specific plasmid types. Further gene-based analyses followed by functional validation will be required to gain improved insights into the molecular factors that are involved in ecological niche adaptation and human pathogenesis.

## Supporting information

S1 Table List of isolates including detailed information. Table includes information about epidemiological metadata, genome completeness, *vls/vlsE* located plasmid, chromosome and plasmid numbers, plasmid presence/absence matrix.
(XLSX)

S2 Table List of PFam32 loci accession numbers. (XLSX)

S3 Table List of plasmid names with differences between published names and names used in this study. (XLSX)

S4 Table Number of genome elements per assembler used in the final combined consensus of the 30 newly sequenced isolates. (XLSX)

S5 Table Detailed information of descriptive statistics of whole dataset, grouped by species, reservoir host, vector and pathogenicity. (XLSX)

S6 Table Results of the GLMMs run on a subset of isolates which either use rodents-only (n = 40) or birds-only (n = 17) as reservoir hosts in relation to total, circular, and linear plasmid counts. The model was run with a Poisson error distribution using the function “glmer” from the R-package lme4 [104]. Mean estimates and their 95% credible intervals (CIs) were estimated based on 10000 simulations using the function “sim” from the package arm [105].
(XLSX)

S7 Table Results of the GLMMs run to test interactions between host associations, vector associations, and human pathogenicity groups and total, circular, and linear plasmid counts of the isolates included in the study (n = 86). The model was run with a Poisson error distribution using the function “glmer” from the R-package lme4 [104]. Mean estimates and their 95% credible intervals (CIs) were estimated based on 10000 simulations using the function “sim” from the package arm [105].
(XLSX)

S1 Fig Schematic overview of the workflow. Lab preparation (grey), PacBio and Illumina sequencing (blue and orange, respectively; combination of PacBio and Illumina data: purple), QC and refinement steps (green) and the generation of a final consensus (red).
(TIF)

S2 Fig Maximum likelihood tree of the 86 genomes based on cgMLST and (fusion) plasmid presence/absence matrix. Figure includes information about species, continent, *vls/vlsE* location, vector, reservoir host, pathogenicity and genome elements (chromosome, plasmids, and fusion plasmids). Species: listed and shown in different colors. Vectors: confirmed vectors are indicated in orange. Reservoir hosts: confirmed reservoir hosts are indicated in dark blue, potential vectors in light blue. Pathogenicity: confirmed status is shown in dark purple; potential pathogenicity is indicated in light purple. Genome elements (chromosome, plasmids, fusion plasmids): presence is indicated green (complete genome element) and red (incomplete genome element), absence is indicated in grey; “X”: fusion plasmid, “-”: linear plasmid that carry a PFam32 that is typically found on a circular plasmid, “O”: circular plasmid that carry a PFam32 that is typically found on a linear plasmid. “$”: cp32-28-4 (circular plasmid with syntenic gene contents to cp32 plasmid family but encodes a lp28−4 type PFam32 protein), “^”: inverted repeat plasmid, “~”: dimer fusion, “+”: sequence was additionally added, “2”: two cp9 plasmids with different PFam57 proteins. “(X)”: the same cp32−11 type PFam32 was found on non-fused plasmid (cp32−11) and fusion plasmid (cp32−9 + 11); “!”: plasmid name used in this study differs to the published plasmid name (S3 Table). S1 Table presents this information in tabular form with additional information on plasmid length.
(TIF)

S3 Fig Reservoir host specific analyses of plasmid numbers per topology and plasmid types. (A) Boxplot showing the spread of plasmid numbers per topology grouped by reservoir host class. Colors correspond to reservoir host class (legend is shown in B). Kruskal-Wallis and pairwise Wilcoxon results are shown in the boxplots. The category total contains linear, circular and fusion plasmids, while linear and circular plasmids contain fusion plasmids. (B) Barplot showing the prevalence of plasmid types per reservoir host class. The length of the bars corresponds to the count of the plasmid type in the total dataset and the exact number is shown at the right tip of the bar. Bars are colored according to the prevalence per reservoir host class and corresponding counts are shown as numbers in the colored bar. (C) Estimated proportions of plasmid presence with 95% confidence intervals of linear (left), circular (middle), and fusion (right) plasmids of “birds-only associated” *vs* “rodents-only associated” isolates (colored in blue and red, respectively, according to legend in B). The two observations are plotted separately above (red: rodents-only) and below (blue: birds-only) the central horizontal line per plasmid type.
(TIF)

S4 Fig Reservoir host specific analyses (multiple *vs* single) of plasmid numbers per topology and plasmid types. (A) Boxplot showing the spread of plasmid numbers per topology grouped by reservoir host class. Colors correspond to reservoir host class (legend is shown in B). Kruskal-Wallis and pairwise Wilcoxon results are shown in the boxplots. The category total contains linear, circular and fusion plasmids, while linear and circular plasmids contain fusion plasmids. (B) Barplot showing the prevalence of plasmid types per reservoir host class. The length of the bars corresponds to the count of the plasmid type in the total dataset and the exact number is shown at the right tip of the bar. Bars are colored according to the prevalence per reservoir host class and corresponding counts are shown as numbers in the colored bar. (C) Estimated proportions of plasmid presence with 95% confidence intervals of linear (left), circular (middle), and fusion (right) plasmids of “single” *vs* “multiple” reservoir hosts isolates (colored in yellow and green, respectively, according to legend in B). The two observations are plotted separately above (yellow: single) and below (green: multiple) the central horizontal line per plasmid.
(TIF)

S5 Fig Vector species specific analyses of plasmid numbers per topology and plasmid types. (A) Boxplot showing the spread of plasmid numbers per topology grouped by vector classes. Colors correspond to vector class (legend is shown in B). Kruskal-Wallis results are shown in the boxplots. The category total contains linear, circular and fusion plasmids, while linear and circular plasmids contain fusion plasmids. (B) Barplot showing the prevalence of plasmid types per vector class. The length of the bars corresponds to the count of the plasmid type in the total dataset and the exact number is shown at the right tip of the bar. Bars are colored according to the prevalence per vector class and corresponding counts are shown as numbers in the colored bar.
(TIF)

S6 Fig Vector specific analyses (multiple *vs* single) of plasmid numbers per topology and plasmid types. (A) Boxplot showing the spread of plasmid numbers per topology grouped by vector class. Colors correspond to vector class (legend is shown in B). Kruskal-Wallis and pairwise Wilcoxon results are shown in the boxplots. The category total contains linear, circular and fusion plasmids, while linear and circular plasmids contain fusion plasmids. (B) Barplot showing the prevalence of plasmid types per vector class. The length of the bars corresponds to the count of the plasmid type in the total dataset and the exact number is shown at the right tip of the bar. Bars are colored according to the prevalence per vector class and corresponding counts are shown as numbers in the colored bar. (C) Estimated proportions of plasmid presence with 95% confidence intervals of linear (left), circular (middle), and fusion (right) plasmids of “single” *vs* “multiple” vector species associated isolates (colored in yellow and green, respectively, according to legend in B). The two observations are plotted separately above (yellow: single) and below (green: multiple) the central horizontal line per plasmid.
(TIF)

S7 Fig Pathogenicity specific analyses of plasmid numbers per topology and plasmid types. (A) Boxplot showing the spread of plasmid numbers per topology grouped by pathogenicity potential. Colors correspond to pathogenicity group (legend is shown in B). Kruskal-Wallis and pairwise Wilcoxon results are shown in the boxplots. The category total contains linear, circular and fusion plasmids, while linear and circular plasmids contain fusion plasmids. (B) Barplot showing the prevalence of plasmid types per pathogenicity group. The length of the bars corresponds to the count of the plasmid type in the total dataset and the exact number is shown at the right tip of the bar. Bars are colored according to the prevalence per pathogenicity group and corresponding counts are shown as numbers in the colored bar. (C) Estimated proportions of plasmid presence with 95% confidence intervals of linear (left), circular (middle), and fusion (right) plasmids of “nonpathogenic” *vs* “pathogenic” isolates (colored in green and red, respectively, according to legend in B). The two observations are plotted separately above (red: pathogenic) and below (green: nonpathogenic) the central horizontal line per plasmid.
(TIF)

S8 Fig Plasmid co-occurrence. Only plasmids with positive (orange) or negative (blue) co-occurrences are shown, while plasmids with only random co-occurrences are not shown. (A) all isolates, (B) pathogenic isolates, (C) single *vs* multiple reservoir host, (D) single *vs* multiple vectors, birds-only *vs* rodents-only.
(TIF)

S1 File Detailed results of plasmid variation and plasmid co-occurrences among different ecological adaptations and pathogenicity groups. (PDF)
