# Supplementary figures and images for: The wild, wild west of plasmids: First insights into comparative genomics of Borrelia burgdorferi sensu lato
Source: PLoS One. 2026 May 8;21(5):e0346097. doi: 10.1371/journal.pone.0346097 (PMC13155636; doi:10.1371/journal.pone.0346097)

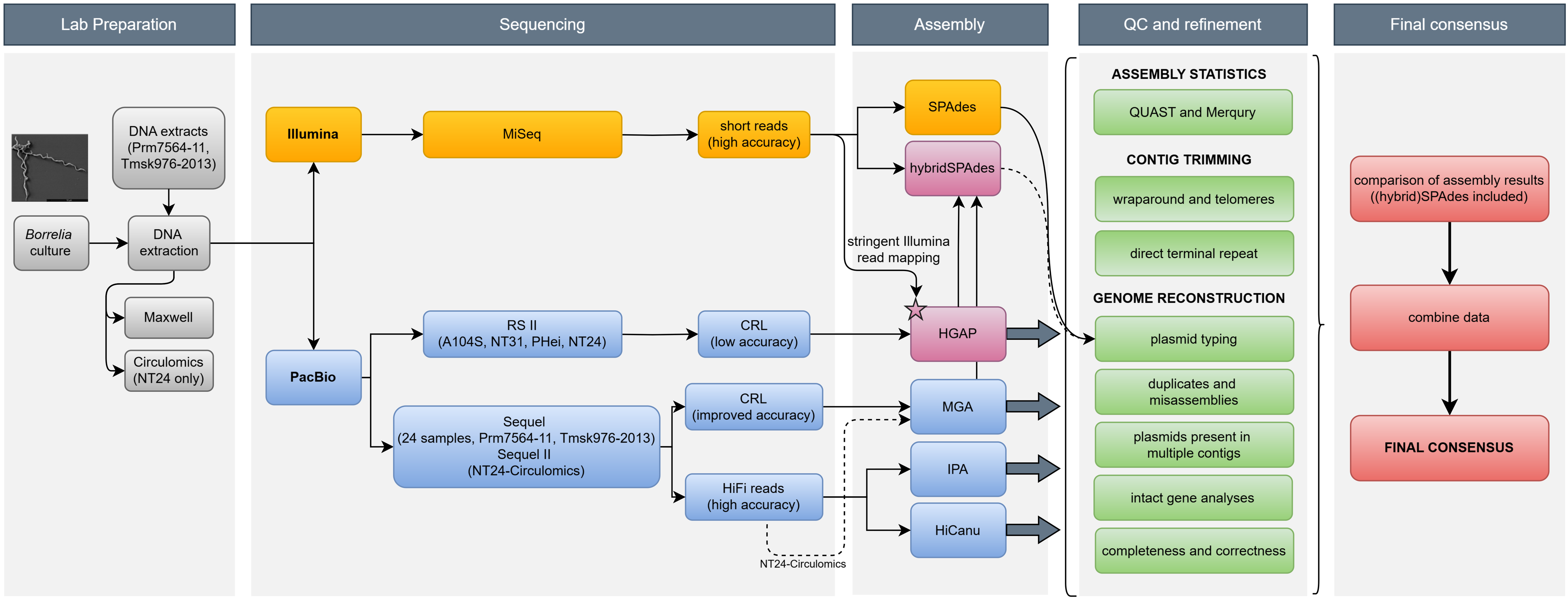

Supplement: S1 Fig — Lab preparation (grey), PacBio and Illumina sequencing (blue and orange, respectively; combination of PacBio and Illumina data: purple), QC and refinement steps (green) and the generation of a final consensus (red). (TIF) [file pone.0346097.s008.tif]

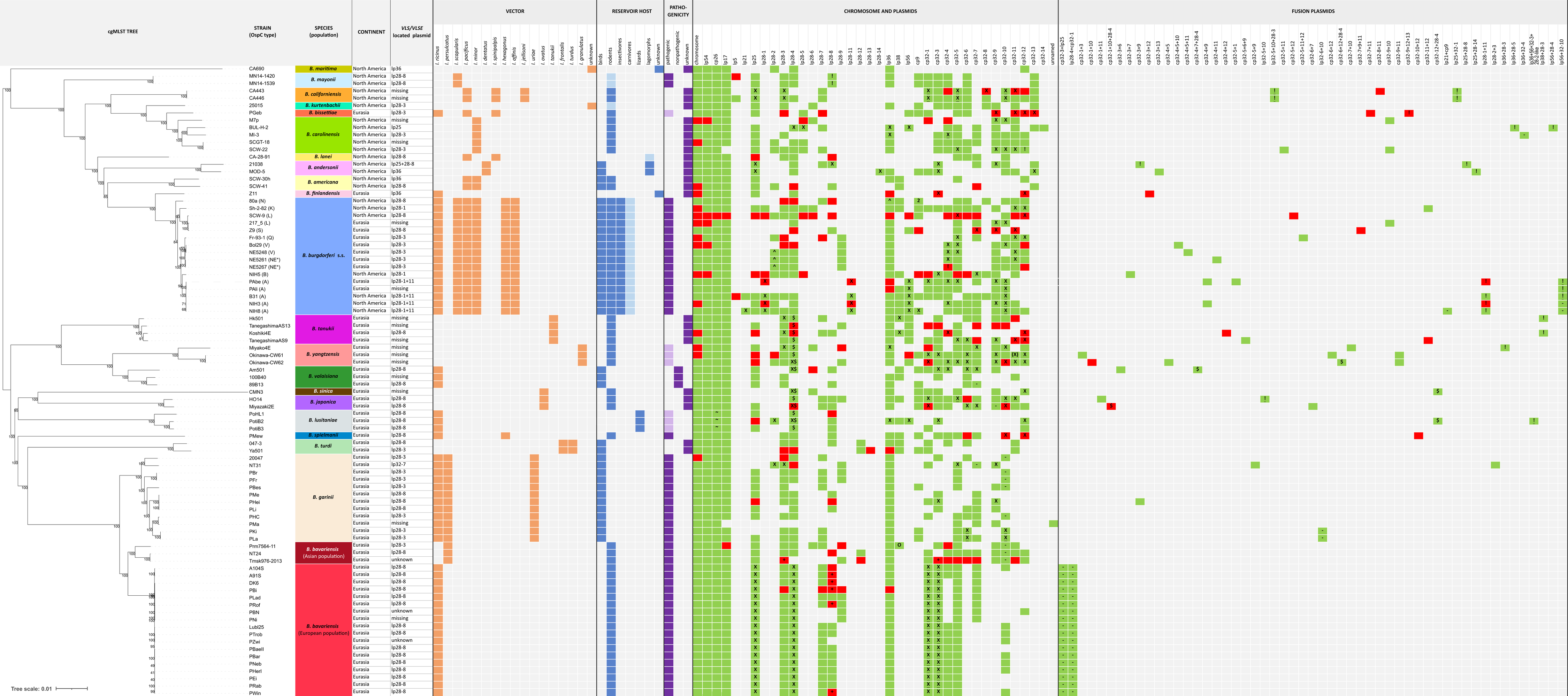

Supplement: S2 Fig — Figure includes information about species, continent, vls/vlsE location, vector, reservoir host, pathogenicity and genome elements (chromosome, plasmids, and fusion plasmids). Species: listed and shown in different colors. Vectors: confirmed vectors are indicated in orange. Reservoir hosts: confirmed reservoir hosts are indicated in dark blue, potential vectors in light blue. Pathogenicity: confirmed status is shown in dark purple; potential pathogenicity is indicated in light purple. Genome elements (chromosome, plasmids, fusion plasmids): presence is indicated green (complete genome element) and red (incomplete genome element), absence is indicated in grey; “X”: fusion plasmid, “-”: linear plasmid that carry a PFam32 that is typically found on a circular plasmid, “O”: circular plasmid that carry a PFam32 that is typically found on a linear plasmid. “$”: cp32-28-4 (circular plasmid with syntenic gene contents to cp32 plasmid family but encodes a lp28−4 type PFam32 protein), “^”: inverted repeat plasmid, “~”: dimer fusion, “+”: sequence was additionally added, “2”: two cp9 plasmids with different PFam57 proteins. “(X)”: the same cp32−11 type PFam32 was found on non-fused plasmid (cp32−11) and fusion plasmid (cp32−9 + 11); “!”: plasmid name used in this study differs to the published plasmid name (S3 Table). S1 Table presents this information in tabular form with additional information on plasmid length. (TIF) [file pone.0346097.s009.tif]

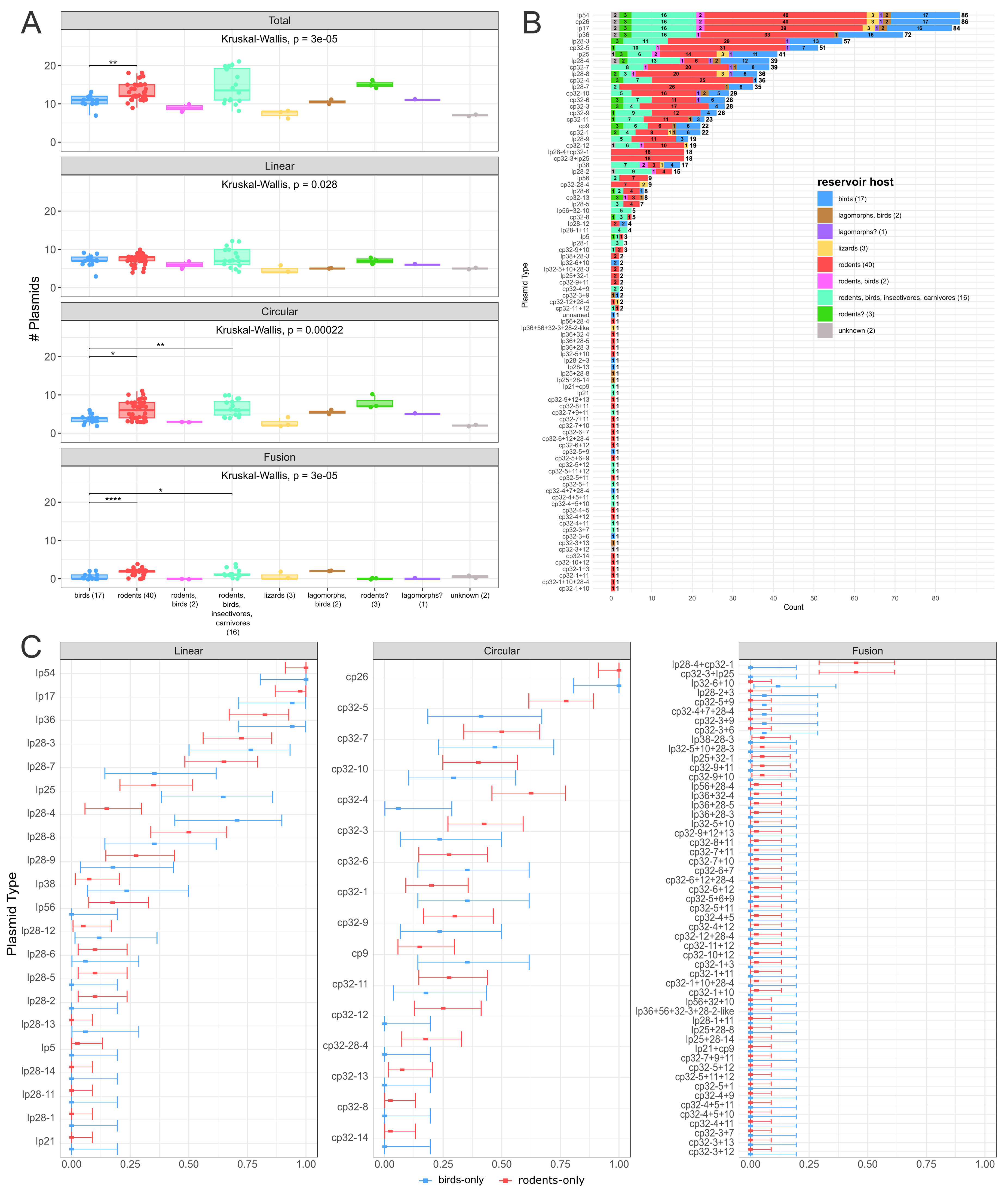

Supplement: S3 Fig — (A) Boxplot showing the spread of plasmid numbers per topology grouped by reservoir host class. Colors correspond to reservoir host class (legend is shown in B). Kruskal-Wallis and pairwise Wilcoxon results are shown in the boxplots. The category total contains linear, circular and fusion plasmids, while linear and circular plasmids contain fusion plasmids. (B) Barplot showing the prevalence of plasmid types per reservoir host class. The length of the bars corresponds to the count of the plasmid type in the total dataset and the exact number is shown at the right tip of the bar. Bars are colored according to the prevalence per reservoir host class and corresponding counts are shown as numbers in the colored bar. (C) Estimated proportions of plasmid presence with 95% confidence intervals of linear (left), circular (middle), and fusion (right) plasmids of “birds-only associated” vs “rodents-only associated” isolates (colored in blue and red, respectively, according to legend in B). The two observations are plotted separately above (red: rodents-only) and below (blue: birds-only) the central horizontal line per plasmid type. (TIF) [file pone.0346097.s010.tif]

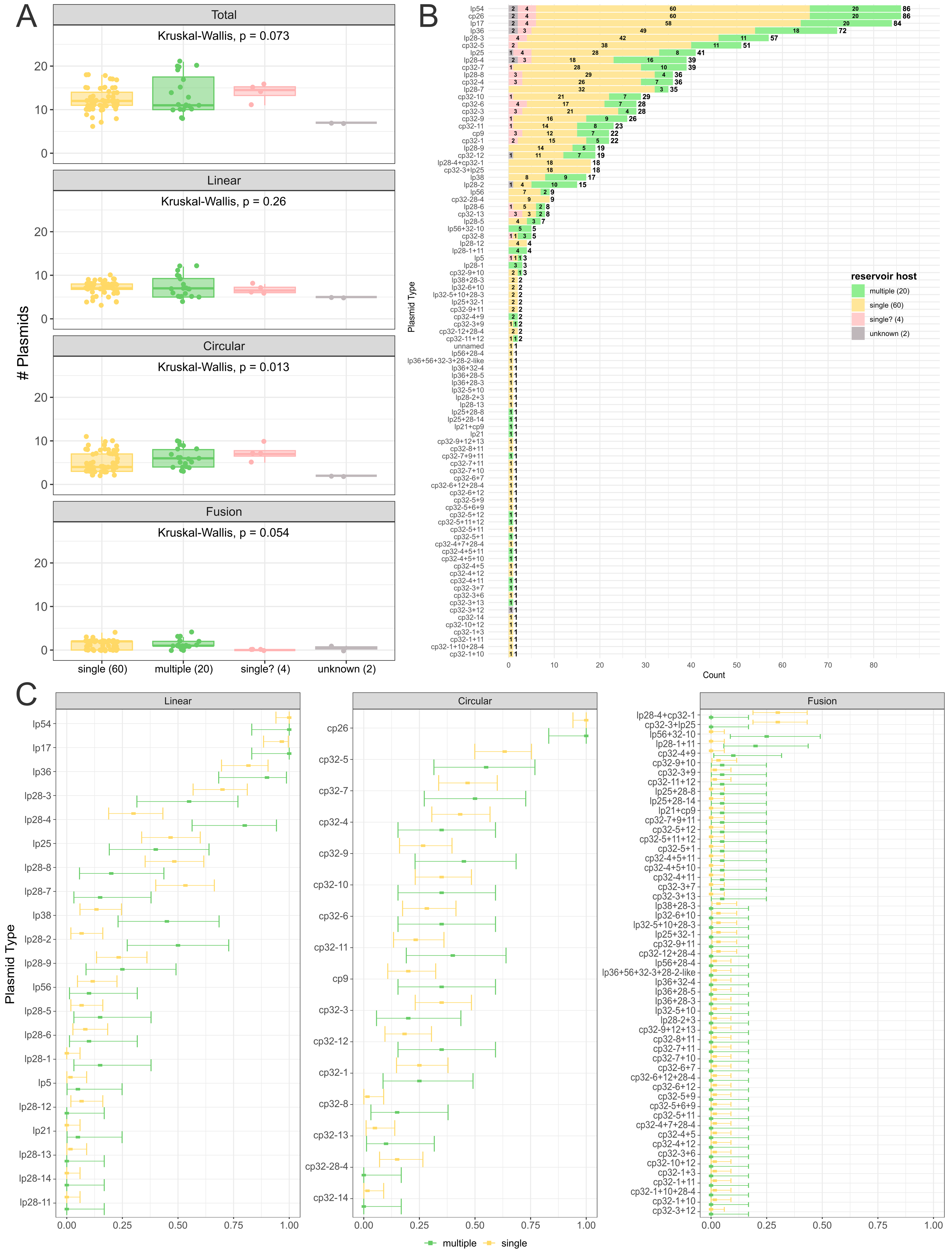

Supplement: S4 Fig — (A) Boxplot showing the spread of plasmid numbers per topology grouped by reservoir host class. Colors correspond to reservoir host class (legend is shown in B). Kruskal-Wallis and pairwise Wilcoxon results are shown in the boxplots. The category total contains linear, circular and fusion plasmids, while linear and circular plasmids contain fusion plasmids. (B) Barplot showing the prevalence of plasmid types per reservoir host class. The length of the bars corresponds to the count of the plasmid type in the total dataset and the exact number is shown at the right tip of the bar. Bars are colored according to the prevalence per reservoir host class and corresponding counts are shown as numbers in the colored bar. (C) Estimated proportions of plasmid presence with 95% confidence intervals of linear (left), circular (middle), and fusion (right) plasmids of “single” vs “multiple” reservoir hosts isolates (colored in yellow and green, respectively, according to legend in B). The two observations are plotted separately above (yellow: single) and below (green: multiple) the central horizontal line per plasmid. (TIF) [file pone.0346097.s011.tif]

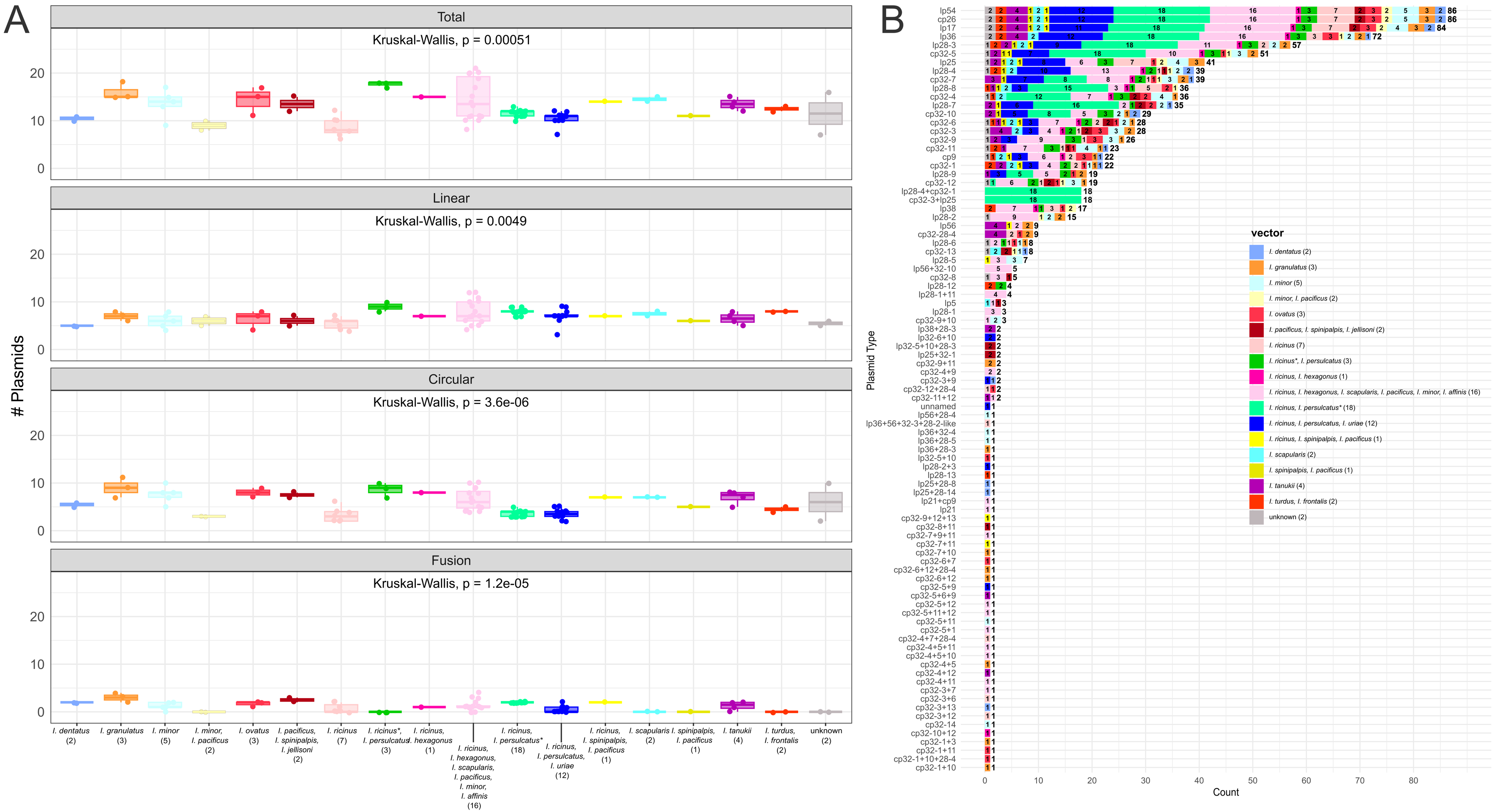

Supplement: S5 Fig — (A) Boxplot showing the spread of plasmid numbers per topology grouped by vector classes. Colors correspond to vector class (legend is shown in B). Kruskal-Wallis results are shown in the boxplots. The category total contains linear, circular and fusion plasmids, while linear and circular plasmids contain fusion plasmids. (B) Barplot showing the prevalence of plasmid types per vector class. The length of the bars corresponds to the count of the plasmid type in the total dataset and the exact number is shown at the right tip of the bar. Bars are colored according to the prevalence per vector class and corresponding counts are shown as numbers in the colored bar. (TIF) [file pone.0346097.s012.tif]

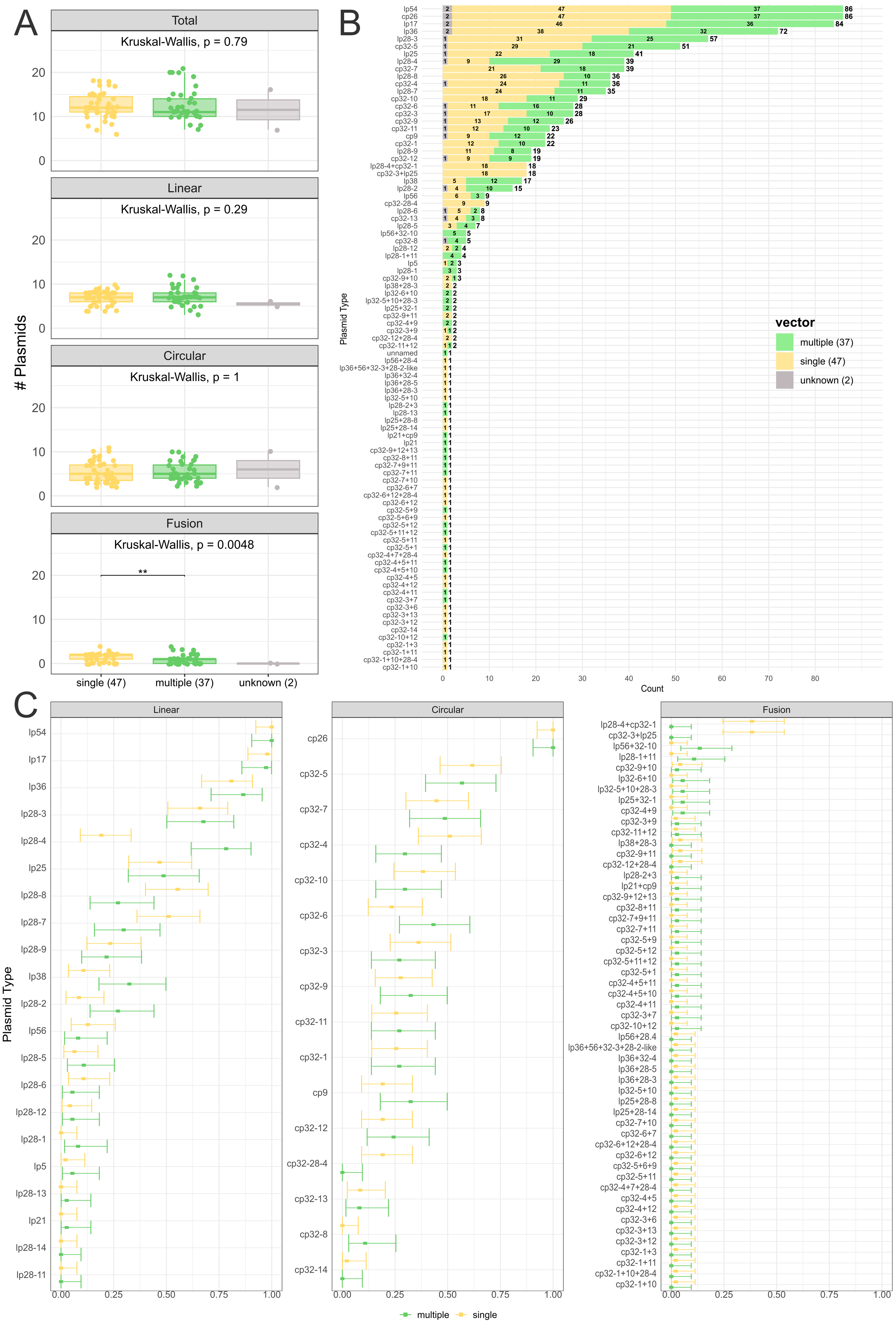

Supplement: S6 Fig — (A) Boxplot showing the spread of plasmid numbers per topology grouped by vector class. Colors correspond to vector class (legend is shown in B). Kruskal-Wallis and pairwise Wilcoxon results are shown in the boxplots. The category total contains linear, circular and fusion plasmids, while linear and circular plasmids contain fusion plasmids. (B) Barplot showing the prevalence of plasmid types per vector class. The length of the bars corresponds to the count of the plasmid type in the total dataset and the exact number is shown at the right tip of the bar. Bars are colored according to the prevalence per vector class and corresponding counts are shown as numbers in the colored bar. (C) Estimated proportions of plasmid presence with 95% confidence intervals of linear (left), circular (middle), and fusion (right) plasmids of “single” vs “multiple” vector species associated isolates (colored in yellow and green, respectively, according to legend in B). The two observations are plotted separately above (yellow: single) and below (green: multiple) the central horizontal line per plasmid. (TIF) [file pone.0346097.s013.tif]

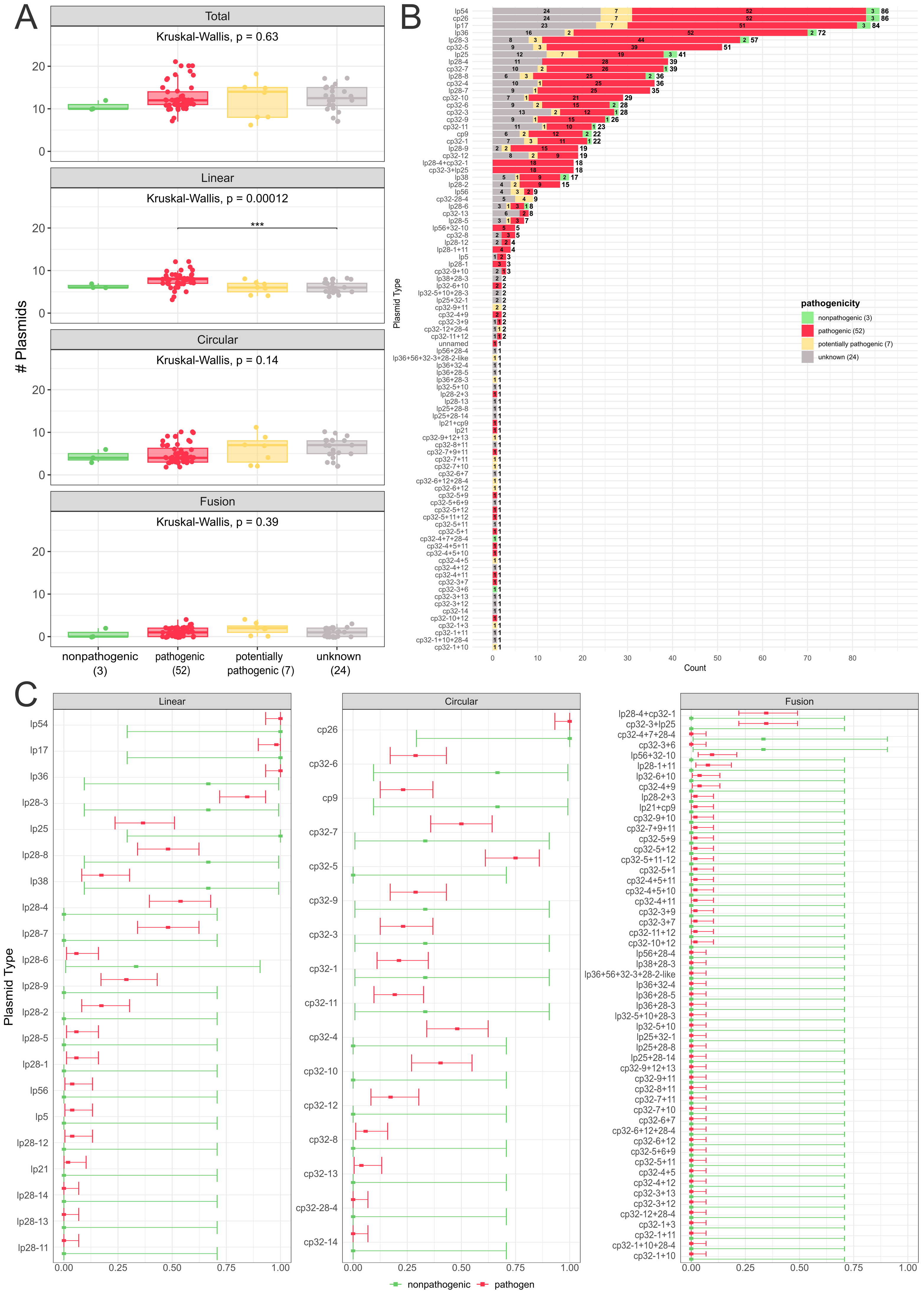

Supplement: S7 Fig — (A) Boxplot showing the spread of plasmid numbers per topology grouped by pathogenicity potential. Colors correspond to pathogenicity group (legend is shown in B). Kruskal-Wallis and pairwise Wilcoxon results are shown in the boxplots. The category total contains linear, circular and fusion plasmids, while linear and circular plasmids contain fusion plasmids. (B) Barplot showing the prevalence of plasmid types per pathogenicity group. The length of the bars corresponds to the count of the plasmid type in the total dataset and the exact number is shown at the right tip of the bar. Bars are colored according to the prevalence per pathogenicity group and corresponding counts are shown as numbers in the colored bar. (C) Estimated proportions of plasmid presence with 95% confidence intervals of linear (left), circular (middle), and fusion (right) plasmids of “nonpathogenic” vs “pathogenic” isolates (colored in green and red, respectively, according to legend in B). The two observations are plotted separately above (red: pathogenic) and below (green: nonpathogenic) the central horizontal line per plasmid. (TIF) [file pone.0346097.s014.tif]

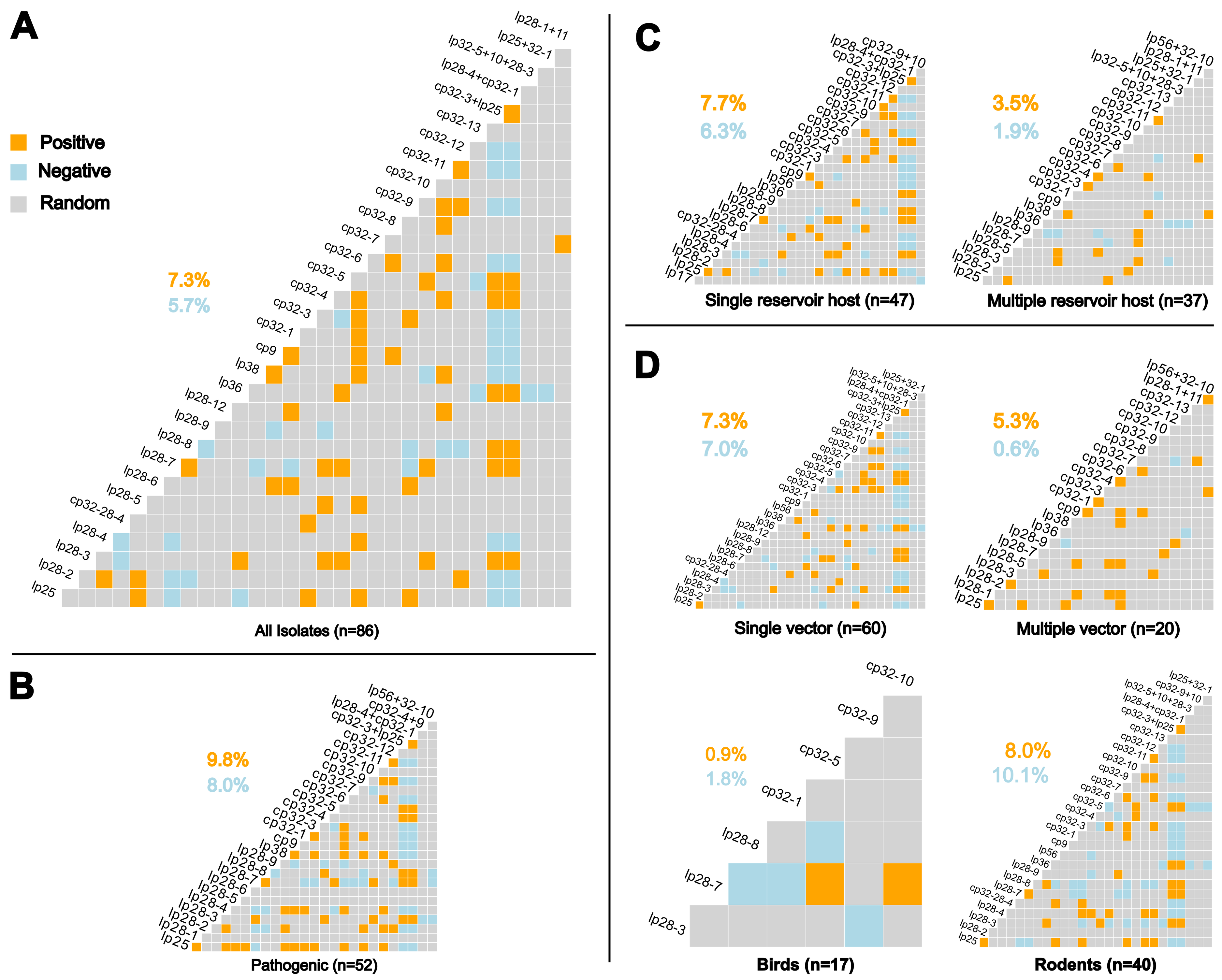

Supplement: S8 Fig — Only plasmids with positive (orange) or negative (blue) co-occurrences are shown, while plasmids with only random co-occurrences are not shown. (A) all isolates, (B) pathogenic isolates, (C) single vs multiple reservoir host, (D) single vs multiple vectors, birds-only vs rodents-only. (TIF) [file pone.0346097.s015.tif]
