# Supplementary material for: The wild, wild west of plasmids: First insights into comparative genomics of Borrelia burgdorferi sensu lato
Source: PLoS One. 2026 May 8;21(5):e0346097. doi: 10.1371/journal.pone.0346097 (PMC13155636; doi:10.1371/journal.pone.0346097)
Supplement: S1 File — (PDF) [file pone.0346097.s016.pdf]

S1 File Hepner et al. 2026

Reservoir host – plasmid relationships

Plasmid numbers – comparison of various reservoir host classes. Plasmid numbers across the various reservoir hosts vary, with significant differences between the groups for total, circular and fusion plasmid numbers (S3A Fig, S5 Table). A significantly higher number of total, circular and fusion plasmids is observed in rodents-only associated isolates (m_{total} = 12.0, $m_{circular}$ = 6.0, m_{fusion} = 2.0) in comparison to birds-only associated ones (m_{total} = 11.0, $m_{circular}$ = 4.0, m_{fusion} = 0.0) ($p.adj_{total}$= 0.002 "**", $p.adj_{circular}$ = 0.04 "*",$p.adj_{fusion}$ = 4.88E-05 "****", see also S6 Tab). We note that potentially rodent-associated isolates ("rodents?", n=3) also have high total and circular plasmid numbers. However, differences are not significant probably due to low sample numbers. Additionally, isolates associated with the reservoir host class "rodents, birds, insectivores, and carnivores" show a significantly higher number of circular and fusion plasmids ($m_{circular}$ = 6.0, m_{fusion} = 1.0) in comparison to birds-only associated isolates ($m_{circular}$ = 4.0, m_{fusion} = 0.0) ($p.adj_{circular}$ = 0.003 "**",$p.adj_{fusion}$ = 0.026 "*"). No significant differences are observed between linear plasmid numbers of the various reservoir host classes.

Linear to circular plasmid numbers ratio in birds-only *vs* rodents-only associated species/population. We observed significantly higher total, circular and fusion plasmid numbers in rodents-only associated isolates compared to bird-only ones (above, S3A Fig, S6 Table). In the whole dataset the number of linear plasmids was significantly higher ($m_{overall\_linear}$=7.0) than circular plasmids ($m_{overall\_circular}$=5.0) (Fig 2A, S5 Table). This observation is also true for birds-only associated isolates (belonging to the species *B. garinii*, *B. turdi*, *B. valaisiana*) (Fig 3). However, we observed a trend of more circular plasmids relative to linear plasmids in rodents-only associated isolates (belonging to the species Asian *B. bavariensis*, *B. bissettiae*, *B. californiensis*, *B. carolinensis*, *B. japonica*, *B. sinica*, *B.*

spielmanii, *B. tanukii*, *B. yangtzensis*) with the exception of the European *B. bavariensis* population (Fig 3).

Plasmid types - comparison of various reservoir host classes. Plasmids lp28-4+cp32-1 and cp32-3+lp25 are exclusively found in rodents-only adapted *B. bavariensis* EU, and plasmids lp56+32-10, lp28-1+11 and lp28-1 are only found in a subset of *B. burgdorferi* s.s. associated with the reservoir host class "rodents, birds, insectivores, and carnivores". No further plasmids that are associated with specific reservoir host classes and that are present in more than two *B. burgdorferi* s. l. isolates are found (S3B Fig). Comparing birds-only associated isolates with the rodents-only associated isolates, analyses of plasmid presence (95% CI) revealed higher frequency of plasmid lp28-4 in birds-only-associated isolates, while plasmids cp32-5, cp32-4, lp28-4-cp32-1, cp32-3+lp25 had higher frequency in rodents-only-associated isolates (S3C Fig). However, it needs to be considered that the lp28-4 PFam32 type is frequently present in both groups: as non-fused plasmids lp28-4 in birds-associated isolates and as fusion plasmid lp28-4+cp32-1 in rodent associated European *B. bavariensis* isolates. Therefore, we only consider cp32-5 and especially cp32-4 as plasmid types that occur notably more often in rodents-only adapted species *vs* birds-only, but since they were also present in the birds-only group no clear group specific association was observed.

Plasmid numbers – comparison of single *vs* multiple reservoir hosts. When isolates currently known to be effectively transmitted by a single (n=60) or by multiple (n=20) reservoir host classes (S1 Table) are compared, no significant differences in the number of plasmids of all topologies are found (Kruskal-Wallis, $p > 0.05$, see also S7 Table). Nevertheless, there is a higher IQR, meaning wide spread of the middle 50% of the data, for total and linear plasmid numbers for isolates associated with multiple reservoir hosts (IQR_{total}=7.5, IQR_{linear}=4.3) compared to a single reservoir host (IQR_{total}=3.0, IQR_{linear}=1.3) (S4A Fig, S5 Table).

Plasmid types - comparison of single *vs* multiple reservoir hosts. The plasmids lp56+32-10, lp28-1, and lp28-1+11 are only found in a subset of the *B. burgdorferi* s.s. isolates and are therefore only found in isolates that are associated with multiple reservoir hosts, while plasmids lp28-4+cp32-1, cp32-3+lp25, cp32-28-4, and lp28-12 are exclusively present in *B. burgdorferi* s.l. species that are associated with a single reservoir host (S4B Fig). The remaining plasmids are only found in one or two isolates. Apart from the previously mentioned plasmids that are only found in one of the groups, a higher frequency of plasmid lp28-8 and lp28-7 was observed in isolates associated with a single reservoir host, while plasmids lp28-4, lp38, and lp28-2 were more frequent in isolates associated with multiple reservoir hosts (S4C Fig). Again, it should be recalled that the PFam32 type of lp28-4 is also frequently present as fusion plasmid lp28-4+cp32-1 in single reservoir host associated isolates (European *B. bavariensis*). In conclusion, no strong convincing correlations were found among plasmid types and reservoir hosts.

Vector – plasmid relationships

Plasmid numbers – comparison of various vector classes and single *vs* multiple vectors. Grouping *B. burgdorferi* s.l. species according to their associated tick vector species show substantial plasmid number variation and significant differences for all plasmid topologies (Kruskal-Wallis, $p < 0.05$) (S5A Fig). In comparison, species associated with single *vs* multiple vector species show no significant difference in total, linear and circular plasmid numbers or in interquartile range (spread of the middle 50% of the data) (S6A Fig, S7 Table). Only the fusion plasmid number is significantly higher in isolates associated with a single vector species compared to multiple ($p.adj_{fusion} = 0.005$, “**”). Thus, in contrast to the results based on single *vs* multiple reservoir hosts association (above), no effect on the spread of plasmid numbers is observed comparing species associated with a single *vs* multiple vectors.

Plasmid types – comparison of various vector classes and single *vs* multiple vectors. The previously described plasmids lp28-4+cp32-1 and cp32-3+lp25 (associated with European *B. bavariensis;* vectored by a single tick species "*I. ricinus*") and lp56+32-10, lp28-1 and lp28-1+11 (associated with a subset of the analyzed *B. burgdorferi* s.s.; vectored by multiple tick species "*I. ricinus, I. hexagonus, I. scapularis, I. pacificus, I. minor, I. affinis*") are exclusively found in one group (S5B and S6B Figs). Additionally, cp32-28-4 was only found in nine isolates vectored by a single tick species. Probability analyses of plasmid presence (95% CI) (S6C Fig) indicate that additionally the non-fused lp28-4 (also excluding cp32-28-4) occurs considerably more frequently in isolates associated with multiple vectors compared to single vector. However, this may be misleading as the PFam32 type of lp28-4 is also present on a fusion plasmid lp28-4+cp32-1 in isolates associated with a single vector. Several further correlations are observed (lp28-8, lp28-7, cp32-4 in single vector associated species; lp38, lp28-2, cp32-6, cp32-8 in multiple associated species) but none seems strong enough to be responsible for vector associations.

Human pathogenicity - plasmid relationships

Plasmid numbers – comparison of pathogenicity groups. Analyses of the plasmid numbers (S7A Fig, S5 and S7 Tables) show mostly no significant differences in plasmid numbers for the groups of differing human pathogenicity (Kruskal-Wallis, $p > 0.05$ for total, circular and fusion plasmid numbers). An exception may be that the linear plasmid number of pathogenic isolates was significantly higher (m = 8.0) than these of isolates with unknown pathogenicity status (m = 6.0) ($p.adj_{linear} = 0.000486$, "***", see also S7 Table). Boxplots (S7A Fig) suggest a trend that the total and linear plasmid numbers may be higher in pathogenic isolates ($m_{total} = 12.0$, $m_{linear} = 8.0$) compared to nonpathogenic isolates ($m_{total} = 10.0$, $m_{linear} = 6.0$). Nevertheless, these

differences are not statistically significant probably due to low sample size of the nonpathogenic isolates.

Plasmid types – comparison of pathogenicity groups. In our analysis, the only plasmids present in a species-specific manner in human pathogenic borreliae are lp28-4+cp32-1 and cp32-3+lp25 (“pathogenic”; European *B. bavariensis*) and lp56+32-10, lp28-1+11 and lp28-1 (“pathogenic”; *B. burgdorferi* s.s.), but they are not present in all pathogenic species (S7B Fig). The remaining plasmids that were exclusively found in isolates belonging to the same pathogenicity group were only present in one or two isolates. We noted that some frequently found plasmids were not present in the nonpathogenic isolates (e.g. the non-fused plasmid cp32-5 is present in 51 isolates but not in nonpathogenic isolates), but this may be due to the low sample size of nonpathogenic isolates.

The probability analyses of plasmid presence (95% CI) confirm these results (S7C Fig). However, those need to be taken cautiously since mostly the range of confidence intervals was overlapping (large CI of nonpathogenic isolates due to low sample number of this category). A noticeable wide range of non-overlapping CI area, indicating a statistically meaningful difference between the compared groups, is observed for plasmid cp32-5 which is more frequently found in pathogenic isolates compared to nonpathogenic. In contrast, the non-fused plasmids lp25 and lp38 show a clear trend of higher frequency in nonpathogenic isolates compared to the pathogenic group, but PFam32 type of lp25 was also present as fusion plasmid in pathogenic isolates (e.g. cp32-3+lp25 in European *B. bavariensis*). Other plasmids show not such clear trends in higher prevalence in pathogenic isolates (e.g. lp36, lp28-4, lp28-7, cp32-4, cp32-10) or in nonpathogenic isolates (e.g. lp28-8, lp28-6, cp32-6, cp9, cp32-4+7+28-4, cp32-3+6). Due to the limited sample numbers of nonpathogenic isolates, these results should be interpreted with caution. Nevertheless, we hypothesize that plasmid cp32-5 may represent an interesting candidate for further gene-based investigation regarding human pathogenicity.

Plasmid co-occurrence

Analyses of plasmid co-occurrence in all isolates (n=86) reveal a positive correlation in 7.3% of the plasmids, while 5.7% of the plasmids have a negative correlation (S8A Figure). Plasmid cp32-6 shows the highest number of positive co-occurrences with other plasmids. In contrast, the fusion plasmids lp28-4+cp32-1 and cp32-3+lp25 have the highest number of negative correlations with other plasmids. These two fusion plasmids are exclusively found in the European *B. bavariensis* isolates that have a reduced variability in the plasmid repertoire, resulting in negative plasmid co-occurrences. Looking only at the pathogenic isolates (n=52) the percentage of plasmids showing positive and negative co-occurrence slightly increased to 9.8% and 8.0%, respectively. A noticeably increase of positive co-occurrences with other plasmids was observed for lp25 when looking at pathogenic isolates compared to all isolates. This was also true for other plasmids, e.g. lp28-4 or lp38. Comparing isolates associated with single *vs* multiple reservoir hosts (S8C Figure) and vectors (S8D Figure), we recognized higher percentage of plasmid correlations (positive and negative) in the isolates associated with a single reservoir host or vector. For example, 7.7% positive and 6.3% negative in single host isolates *vs* 3.5% positive and 1.9% negative in multiple hosts isolates. An even clearer difference was found comparing birds-only *vs* rodents-only adapted isolates. Plasmids of the rodents-only adapted isolates showed 8.0% positive and 10.1% negative correlation with other plasmids (S8D Figure), while we only found 0.9% positive and 1.8% negative plasmid co-occurrence in the birds-only adapted isolates.
